# Small regulatory RNAs modulate lactococcal susceptibility to cell wall-targeting antimicrobials

**DOI:** 10.1093/nar/gkag359

**Published:** 2026-04-25

**Authors:** Milda Mickutė, Kotryna Kvederavičiūtė, Janina Ličytė, Renatas Krasauskas, Sigita Grigaitytė, Oskaras Safinas, Danguolė Žiogienė, Naglis Mykolas Pakštys, Loreta Stankevičiūtė, Algirdas Kaupinis, Mindaugas Valius, Pascal Courtin, Marie-Pierre Chapot-Chartier, Saulius Kulakauskas, Giedrius Vilkaitis

**Affiliations:** Institute of Biotechnology, Life Sciences Center, Vilnius University, Vilnius, LT-10257, Lithuania; Institute of Biotechnology, Life Sciences Center, Vilnius University, Vilnius, LT-10257, Lithuania; Institute of Biotechnology, Life Sciences Center, Vilnius University, Vilnius, LT-10257, Lithuania; Institute of Biotechnology, Life Sciences Center, Vilnius University, Vilnius, LT-10257, Lithuania; Institute of Biotechnology, Life Sciences Center, Vilnius University, Vilnius, LT-10257, Lithuania; Institute of Biotechnology, Life Sciences Center, Vilnius University, Vilnius, LT-10257, Lithuania; Institute of Biotechnology, Life Sciences Center, Vilnius University, Vilnius, LT-10257, Lithuania; Institute of Biotechnology, Life Sciences Center, Vilnius University, Vilnius, LT-10257, Lithuania; Institute of Biotechnology, Life Sciences Center, Vilnius University, Vilnius, LT-10257, Lithuania; Institute of Biochemistry, Life Sciences Center, Vilnius University, Vilnius, LT-10257, Lithuania; Institute of Biochemistry, Life Sciences Center, Vilnius University, Vilnius, LT-10257, Lithuania; Université Paris-Saclay, INRAE, AgroParisTech, Micalis Institute, Jouy-en-Josas, 78352, France; Université Paris-Saclay, INRAE, AgroParisTech, Micalis Institute, Jouy-en-Josas, 78352, France; Université Paris-Saclay, INRAE, AgroParisTech, Micalis Institute, Jouy-en-Josas, 78352, France; Institute of Biotechnology, Life Sciences Center, Vilnius University, Vilnius, LT-10257, Lithuania

## Abstract

The contribution of small RNAs to the regulation of cell wall defence mechanisms—crucial for protecting bacteria from environmental insults—remains poorly understood in *Lactococcus cremoris*, a model organism for studying lactic acid bacteria. Characterization of the profiles of 193 putative small regulatory RNAs (sRNAs), including 131 newly identified in this study, revealed altered expression of 20 and 25 sRNAs following treatment with cell wall-targeting antimicrobials, lysozyme and penicillin G, respectively. Further analysis and genome-wide functional screening identified four sRNAs that affected lysozyme response in opposite directions: elevated levels of sLLM1042+, sLLM2-, and sLLM1993+ were associated with increased resistance, whereas sLLM461+ with decreased resistance. Moreover, elevated expression of sLLM2-, sLLM1042+, or both promoted sensitivity to penicillin G and certain abiotic stresses, and exerted pleiotropic effects on gene regulation at both RNA and protein levels, suggesting broader regulatory impacts. Notably, the sLLM2- mediated decrease in four putative tellurium resistance proteins resulted in increased sensitivity to tellurite stress. As sLLM2- mutations, altering lysozyme and penicillin responses, also reduced alanine racemase Alr expression, we suggest that sRNA upregulation modulates D-alanine levels, thereby affecting lipoteichoic acid modification or peptidoglycan synthesis. Our findings support a contribution of sRNAs to LAB responses to cell wall-targeting antimicrobials.

## Graphical Abstract

**Graphical Abstract:**
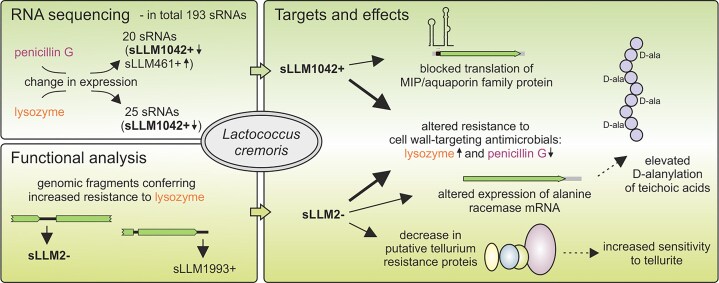

## Introduction

The industrially important bacteria *Lactococcus lactis* and *Lactococcus cremoris* are broadly used in fermentation and food preservation and contribute to the texture and flavour of fermented products [1]. They are currently applied as microbial factories to produce lactic acid, a crucial component for the biodegradable plastics industry, and the natural food preservative nisin, a potential alternative to traditional antibiotics. These bacteria also exert inherent beneficial health effects [2–4] and are increasingly tested as vectors expressing peptides and proteins for oral or intranasal administration to combat viral, bacterial, and parasitic infections, cancer, allergy, inflammation, autoimmune diseases, and metabolic disorders [5, 6]. The cell wall plays a crucial role in withstanding environmental threats, such as exposure to cell wall-targeting antimicrobials, that lactococci encounter during these applications, highlighting the need to understand its response to such stressors. Lysozyme, a 14.7 kDa protein that hydrolyses the β-1,4-glycosidic bond between N-acetylmuramic acid and N-acetylglucosamine, is a common stress factor faced by bacteria in the human body, as it disrupts the cell wall’s peptidoglycan (PG) layer. This antimicrobial enzyme is also widely used in medicine, the food industry, and agriculture [7]. Another group of stressors, β-lactam antibiotics, including penicillin G (PnG), are among the most broadly used antimicrobials worldwide. They are characterized by the β-lactam ring in their structure and target penicillin-binding proteins, which play a key role in transpeptidation during the PG formation. Their β-lactam ring irreversibly binds to penicillin-binding proteins, resulting in weakly crosslinked PG and rendering the bacteria highly susceptible to cell lysis [8]. Despite extensive knowledge of protein-based defence strategies against cell wall-targeting antimicrobials, there is scarce information about the role of small regulatory RNA (sRNA)-mediated networks in these processes.

Over the past decade, investigations have shown that bacteria are able to cope with stress conditions, in part through the action of sRNAs that post-transcriptionally regulate gene expression. It has been suggested that sRNAs may regulate approximately half of the bacterial messenger RNAs (mRNAs) [9], highlighting their significant potential to play important roles in controlling antimicrobial response pathways. Indeed, it has been reported that sRNAs can be involved in modulating antibiotic uptake and efflux, membrane permeability systems, cell envelope modification, and inducing biofilm formation [10]. Nevertheless, much of the current understanding of RNA-based regulation in antimicrobial responses stems from studies on Gram-negative bacteria such as *Escherichia coli* and *Salmonella*. Increasing evidence, however, shows that small RNAs also contribute to the regulation of antibiotic resistance in Gram-positive species, including *Staphylococcus aureus* [11–13] and *Enterococcus faecium* [14, 15]. Expanding such studies in Gram-positive bacteria, such as *Lactococcus*, is essential to further elucidate the RNA-mediated mechanisms underlying antimicrobial resistance.

Our study focuses on the identification and analysis of sRNAs responsible for cell wall adaptation to stress caused by antimicrobials affecting the cell wall integrity, specifically lysozyme and penicillin G, in the model lactic acid bacterium (LAB) *Lactococcus cremoris* subsp. *cremoris* MG1363 (formerly classified as *Lactococcus lactis* subsp. *cremoris* MG1363). To decipher the response to stressors, we analysed genome-wide changes in sRNA profiles following antimicrobial treatment. In parallel, functional screening identified *L. cremoris* genome fragments altering susceptibility to antimicrobials affecting cell wall integrity. Two small RNAs, sLLM2-, and sLLM1042+, were then selected for further analysis, which suggested that they modulate complex sRNA-based networks associated not only with responses to lysozyme and penicillin G, but also with other cellular processes.

## Materials and methods

### Bacterial growth, biomass collection, and RNA purification

*L. cremoris* MG1363 was grown at 30°C in BD Difco™ M17 broth supplemented with 0.5% glucose (GM17) and 5 µg/ml of chloramphenicol when transformed. A complete list of bacterial strains and plasmids used throughout this study is provided in Supplementary Tables S1 and S2, respectively. For biomass collection, the overnight cultures were diluted to an optical density at 600 nm (OD_600_) 0.05–0.1, grown until the selected growth stage, mixed with 1/8 volume of ice-cold STOP solution (95% ethanol, 5% acid phenol), centrifuged at 1 800 × *g* for 20 min at 4°C, and stored at −80°C. Cells were ground to a fine powder using liquid nitrogen, and the total RNA was extracted with RNAzol^RT^ (Molecular Research Center, Inc.) according to the manufacturer’s recommendations. Alternatively, bacteria were suspended in RNAzol^RT^ to a final OD_600_ of up to 20, homogenized twice for 40 s at 5 m/s using an MP Biomedicals homogenizer and processed according to the manufacturer’s recommendations.

### *L. cremoris* sRNA library preparation

For the five-point library, the biomass was collected at OD_600_ 0.2, 0.8, 1.9, 3.0, and 3.3. For treated libraries, bacteria were grown until they reached an OD_600_ 0.5. At this point, penicillin G (PnG) or lysozyme was added to a final concentration of 0.125 µg/ml and 0.5 mg/ml, respectively. Bacteria were further grown to the mid-exponential phase (OD_600_ 1.6–1.8) and collected as described earlier. The quality of the extracted RNA was evaluated using the Agilent RNA 6000 Nano Kit (Agilent Technologies), with all samples achieving an RNA Integrity Number (RIN) value of 9.6–10. For sRNA library preparation, 50–500 nt RNA was cut out of 8% denaturing polyacrylamide gel and further processed as described in [16] with the following modifications: it was treated with DNase I, RNase-free (Thermo Scientific), and recovered from the reaction using RNA Clean & Concentrator-5 Kit (Zymo Research). 5S ribosomal RNA (rRNA) was depleted using the modified RiboMinus Transcriptome Isolation Kit, bacteria (Invitrogen), where 4 μl of 100 μM mix of three probes specific to *L. cremoris* 5S rRNA was used (Supplementary Table S3). After ethanol precipitation, RNA was treated with RNA 5′ pyrophosphohydrolase (RppH) (New England Biolabs), recovered with RNA Clean & Concentrator-5 Kit, and 165 ng were taken for further processing using NEXTflex Small RNA-Seq Kit v3 (PerkinElmer) with the following modifications: at Step E, incubation at 42°C was extended to 60 min; at Step G, 16 polymerase chain reaction (PCR) cycles were performed; and Steps F and H1 were carried out as described in the alternative protocol for Preparing Libraries without Size Selection. The resulting complementary DNA (cDNA) libraries were quantified by KAPA™ Library Quantification Kit (Roche) and sequenced on the Illumina MiSeq™ instrument using MiSeq™ Reagent Kit v2 chemistry (Illumina) for paired-end sequencing with 2 × 151 bp reads.

### sRNA sequencing data analysis

Sequencing data quality was evaluated using FASTQC v0.11.9 program (https://www.bioinformatics.babraham.ac.uk/projects/fastqc/). UMI sequences were removed using UMI-tools v1.1.5 [17]. Low-quality bases and reads were removed using cutadapt v4.4 [18] and SeqPurge v2022_12 [19]. Mapping was performed using hisat2 v2.2.1 [20] and *L. cremoris* MG1363 as a reference sequence (NCBI: NC_009004), manipulations with SAM and BAM files were performed using samtools 1.12 [21].

For potential sRNA prediction, multi-mapping reads were mapped to the leftmost genome position, and deduplication was performed using the “unique” method from the UMI-tools package. Deduplicated reads were then remapped to the genome using hisat2. Finally, APERO [22] with five different parameter sets was used to identify a list of potential sRNAs. Short (<40 bp) and long (>1 kb) sequences were removed from further analysis. Next, sequences without a sharp drop in coverage at the 3′ or 5′ end of the sequence were removed. After that, sequences overlapping with protein or known structural RNAs (70% coverage threshold was used) were also removed from the list. We finally employed a sliding window to evaluate the steepness at the 3′ and 5′ ends of potential sRNAs and selected the final list of sRNAs. All manipulations with sRNAs were performed using R (4.3.0) (https://www.R-project.org).

For the differential expression analysis, all genes and sRNAs were quantified using the featureCounts program from the SubRead v2.0.6 package [23]. Differential gene expression analysis was performed using the RUVr from the RUVseq package [24] using upper-quartile normalization. Log_2_ fold change (Log_2_FC) thresholds of 0.8 and Benhamini–Hochberg ≤ 0.1 were applied to identify differentially expressed sRNAs.

sRNA promoters were identified using BPROM [25] and Promotech [26]. Terminators were predicted using termPred [25] and ARNold [27]. The secondary structure was calculated using RNAfold from the ViennaRNA package [28, 29]. Similarity and sRNAs identification in various bacteria was performed using BLAST 2.9.0+ [30] and Needleman–Wunsch from EMBOSS package [31].

Sequences were aligned using MUSCLE [32] and visualized using JalView [33]. Names were assigned to sRNAs using a modified BSRD-style nomenclature, as detailed in the Supplementary Notes.

### Northern hybridization

Northern hybridization was performed as described in [16] using 7–10 µg of total RNA per lane. For sRNA overexpression and sLLM1042+m[30–33] analysis, total RNA was purified from the biomass collected at the mid-exponential phase. For M-31 and 47–11 deletion analysis, biomass was collected at four growth points (OD_600_ of 0.2, 1, 2.2, and 2.5). Where needed, the relative amount of sRNA was evaluated after its signal intensity was divided by the signal of 5S rRNA. All oligonucleotides used are listed in Supplementary Table S4.

### Plasmid construction

For the psLLM1042+ and psLLM461+ plasmids, the sLLM1042+ or sLLM461+ encoding genes, along with their surrounding areas, were PCR-amplified using sLLM1042+_Fw/sLLM1042+_Rv or sLLM461+_Fw/sLLM461+_Rv primer pairs, respectively, from *L. cremoris* MG1363 genomic DNA (gDNA). The phosphorylated PCR products were ligated into the pVE3916 plasmid, digested with HindIII and XhoI (all restriction endonucleases used in this work were bought from Thermo Scientific), and then blunt-ended.

The M-31-D1 plasmid was made by re-ligating the M-31 plasmid, which was cut with Van91I and XhoI and subsequently blunted with T4 DNA polymerase (Thermo Scientific). M-31-D2 was constructed by re-ligating the M-31 plasmid after digestion with PvuII. For M-31-D3/psLLM2-, the M-31 fragment, amplified using primer pair M-31-tg-dir/M-31-tg-rev, was digested with HindIII and XhoI and ligated into pVEA1 plasmid cut with the same restriction endonucleases. To prepare M-31-D4, the blunted XagI and XhoI fragment of the M-31 was re-ligated back to itself. M-31-D5 was made by re-ligating the M-31 PvuII and XagI fragment, blunted with T4 DNA polymerase. For M-31-D6, the M-31 fragment amplified with primer pair M-31-Δtg-dir/M-31-Δtg-rev was inserted into pVEA1, which was cut with XhoI and blunted with T4 DNA polymerase.

To prepare 47-11-D1, 47-11-D2, and 47-11-D3, FaqI and XhoI, XbaI and FaqI, BseLI and XhoI restriction endonucleases, respectively, were used to digest the 47-11 plasmid. The resulting plasmids were blunted with T4 DNA polymerase and then self-ligated. For 47-11-D4/psLLM1993+, a fragment was amplified from *L. cremoris* MG1363 gDNA using primer pair 47-11-tg-dir/47-11-tg-rev, cut with HindIII and XhoI, and ligated into the pVEA1 plasmid, which was previously digested with the same restriction endonucleases.

For all plasmids containing mutated sLLM2- sequences, psLLM2- vector was digested with MunI and PvuI restriction endonucleases and ligated with MunI or PvuI pre-cut and phosphorylated PCR fragments. For psLLM2-m[12–16] and psLLM2-m[66–69], PCR fragments were obtained using psLLM2- as a template and the following primer pairs: for sLLM2-m[12–16] - sLLM2-mut_Fw1/sLLM2-mut_Rv1 and sLLM2-mut_Fw2/sLLM2-mut_Rv2, for sLLM2-m[66–69] - sLLM2-mut_Fw1/sLLM2-mut_Rv3 and sLLM2-mut_Fw3/sLLM2-mut_Rv2. For plasmid containing sLLM2-m[12–16][66–69], psLLM2-m[12–16][66–69], the first half of sRNA was amplified from psLLM2-m[12–16] using sLLM2-mut_Fw1/sLLM2-mut_Rv4 primer pair and the second half was amplified from psLLM2-m[66–69] using sLLM2-mut_Fw4/sLLM2-mut_Rv2.

For MS2 affinity purification coupled with RNA sequencing (MAPS) experiment, pNis-sLLM1042+ was constructed by amplifying pMSP3545 using primer pair F_invert_plasmid/R_invert_plasmid and ligating semi-bluntly via PaeI with sLLM1042+ that was amplified from gDNA of *L. cremoris* using primer pair sLLM1042+_Fw2/R_LLM_1042_PaeI. pNis-2×MS2-sLLM1042+ was constructed similarly, except primer R_invert_MS2_plasmid was used instead of R_invert_plasmid. To construct pGEX-5X-1-2xMCP, pGEX-5X-1 was cut with SmaI restriction endonuclease and ligated with 2×MCP fragment amplified from phage – ubc – nls – 2xmcp – egfp – BirA* and pre-cut with NdeI and XbaI.

To construct psLLM1042+m[30–33], a modified sRNA sequence was generated using two primer pairs—sLLM1042+mut_Fw1/sLLM1042+mut_Rv1 and sLLM1042+mut_Fw2/sLLM1042+mut_Rv2—with psLLM1042+ as a template. The resulting PCR fragments were digested with TauI and NcoI, phosphorylated, and ligated into psLLM1042+, which had been pre-cut with the same restriction endonucleases.

For GFP reporter assay, to construct pVE-P32-glpF3-GFP+, the *glpF3* gene fragment was amplified from *L. cremoris* gDNA using the primer pair Glp3 dir/glp-GFP+ rev. The GFP+ gene was amplified from the plasmid pZEP16 using the primers glp-GFP+ dir and GFP+ rev. These fragments were joined by overlap extension PCR using Phusion Plus polymerase and the primer pair Glp3 dir/GFP+ rev. The resulting fragment was ligated semi-bluntly via Bsu15I into pVE-P32 pre-digested with PaeI and Bsu15I. pNis-sLLM1042+m[30–33] was constructed by PCR amplification of pMSP3545 using the primer pair F_invert_plasmid/R_invert_plasmid. The resulting fragment was ligated semi-bluntly via PaeI with sLLM1042+m[30–33], which was amplified from psLLM1042+m[30–33] using the primer pair sLLM1042+ Dir/sLLM1042+ Pae Rev.

All oligonucleotides used for plasmid construction are listed in Supplementary Table S5.

### Preparation of electrocompetent *L. cremoris* cells and electroporation

Overnight cultures were diluted 40-fold and grown in GM17 medium with 0.5 M saccharose and 2% glycine at 30°C to an OD_600_ of 0.3–0.6. The cells were then centrifuged at 4000 × *g* for 10 min at 4°C, washed three times with ice-cold 0.5 M saccharose and 10% glycerol, resuspended in 2 ml 30% PEG 3000 with 10% glycerol, and stored in 100 µl aliquots at −80°C. Electrocompetent bacterial cells were mixed with 0.1–1 µg of plasmid DNA and pulsed by BTX Electro Cell Manipulator set at 2.5 kV, 25 μF, 400 Ω. The cells were recovered in 1 ml of GM17 medium at 30°C for 2–4 h, and then plated on GM17 agar supplemented with 5 µg/ml chloramphenicol.

### Assessment of antimicrobial susceptibility on agar plates and in broth culture

For analysis on agar plates, overnight cultures were serially diluted (10-fold) from an OD_600_ of 1 to 10^−5^, spotted onto M17 agar supplemented with 0.5% glucose or 0.5% galactose, 5 µg/ml chloramphenicol, indicated concentrations of antimicrobials, and 0.5 mM cysteine or 2 mM D-alanine where needed, and grown for up to 40 h at 30°C. Each experiment included 2–3 independent biological replicates.

For growth curve analysis, mid-exponential phase bacteria cultures were diluted to OD_600_ of 0.05–0.1 and grown in 200 µl of M17 liquid medium with 5 µg/ml chloramphenicol supplemented with 0.5% glucose, galactose, or cellobiose and indicated concentrations of antimicrobials in 96-well plates using BioTek Synergy H4 plate reader for 10–16 h at 30°C. OD_600_ was measured every 10 min after up to 1 min of shaking. Each experiment included 2–3 independent biological replicates.

For growth curve analysis in a defined minimal liquid medium, bacterial OD_600_ was measured at hourly intervals. A modified BL medium was prepared according to [34] with arginine, serine, and tryptophan, but without (NH_4_)_6_Mo_7_O_24_, and was supplemented with 1 ng/ml nisin and 10 µg/ml erythromycin.

### Construction of a gene bank of *L. cremoris* and selection of the resistant clones

The gDNA of *L. cremoris* was sonicated to generate fragments primarily ranging in size from 300 to 700 bp. Two hundred to one thousand base pair fragments were purified from an agarose gel using GeneJET Gel Extraction Kit (Thermo Scientific) and ligated into a pVEA1 vector (the derivative of pVE3916, containing an additional 1008 bp PagI fragment carrying the ampicillin-resistance gene from the pUC19 plasmid, which was inserted into the XceI site that has been blunted by T4 polymerase), which had been pre-cut with XhoI restriction endonuclease and blunted afterwards. After transforming *E. coli* TG1 with ligation mix, plasmid libraries were purified using GeneJET Plasmid Miniprep Kit (Thermo Scientific) and subsequently used for the electroporation of *L. cremoris* MG1363 or its Δ*oatA* strain. DNA fragments conferring resistance to lysozyme were selected by growing the electroporated cells on selective GM17 agar medium supplemented with 3 mg/ml lysozyme for the wild type (WT) or 1 mg/ml lysozyme for the Δ*oatA* strain at 30°C. The plasmids were then purified using a slightly modified GeneJET Plasmid Miniprep Kit, as 10 mg/ml of lysozyme was added to the Resuspension buffer, and used for another round of electroporation and selection.

### Analysis of D-alanylation of teichoic acid

D-Ala esterification of teichoic acids in *L. cremoris* strains was analysed by high-performance liquid chromatography as previously described [35]. Briefly, lyophilized whole heat-inactivated bacteria were treated with 0.1 N NaOH for 1 h at 37°C to release D-Ala, which was then derivatized with Marfey’s reagent (1-fluoro-2,4-dinitrophenyl-5-L-alanine amide; Sigma). This reagent reacts with the optical isomers of amino acids to form diastereomeric N-aryl derivatives. The amino acid derivatives were separated on a reversed-phase column (Zorbax Eclipse Plus C18 RRHD 2.1 × 50 mm 1.8 µm Agilent) using an Agilent UHPLC 1290 system with a linear elution gradient of acetonitrile in 20 mM sodium acetate buffer, pH 5.0, and UV detection at 340 nm. Quantification was achieved by comparison with D-Ala standards in the range of 50–1000 pmol. Mean values were obtained from three independent cultures.

### RNA library preparation for total RNA sequencing and data analysis

*L. cremoris* MG1363 strain, containing pVE3916, psLLM2-, or psLLM1042+, was grown in liquid GM17 medium with 5 µg/ml chloramphenicol to OD_600_ 0.8. The biomass was collected, and RNA was extracted as described for sRNA library preparation. Total RNA was treated with DNase I, RNase-free, and purified using RNA Clean & Concentrator-5 Kit. rRNA was depleted using the modified RiboMinus Transcriptome Isolation Kit, bacteria, where 3.5 μl of commercial probes and 0.5 μl of a 100 μM mix of three custom-designed probes specific to *L. cremoris* 5S rRNA were used (Supplementary Table S3). After ethanol precipitation, libraries were further prepared in Thermo Fisher Scientific facilities using Invitrogen™ Collibri™ Stranded RNA Library Prep Kit for Illumina. cDNA libraries were sequenced with Illumina NovaSeq 6000 with a read length of 2 × 151 bp.

Total RNA sequencing data analysis was performed similarly as for sRNA: data quality was evaluated using FASTQC v0.11.9 program (https://www.bioinformatics.babraham.ac.uk/projects/fastqc/) and trimmed using BBDUK (https://sourceforge.net/projects/bbmap/). Reads were mapped using hisat2 v2.2.1 [20] and *L. cremoris* MG1363 as a reference sequence (NCBI: NC_009004). StringTie [36] was used to perform quantification. Differential gene expression analysis was performed using edgeR [37]. Enrichment analysis was performed with clusterProfiler package [38] against KEGG database [39].

### RT-qPCR analysis

Sequencing results were validated using the same total RNA. The expression of sLLM2-, its mutants, and *alr* were determined after the collection of bacterial biomasses at the mid-exponential growth phase (OD_600_ of 0.8). Total RNA (500 ng) was treated with DNase I, RNase-free, and used for cDNA synthesis with RevertAid RT reverse transcriptase (Thermo Scientific). Quantitative PCR was performed on a Rotor-Gene 6000 (Corbett Life Science) with SYBR™ Green PCR Master Mix (Thermo Fisher Scientific), according to the manufacturer’s recommendations. Specifically, 250- to 500-fold diluted cDNA, 0.3–0.9 μM of primers (Supplementary Table S6) were used, and the following cycling conditions were applied: initial denaturation for 10 min at 95°C, three cycles of amplification for 15 s at 95°C, 1 min at 55°C, 30 s at 60°C, and 32 cycles of 10 s at 95°C, 30 s at 60°C. For sLLM1042+ deletion verification and enrichment analysis, the cycling conditions were as follows: initial denaturation for 10 min at 95°C, 40 cycles of 15 s at 95°C, 30 s at 60°C, and 30 s at 72°C. The results were calculated from three biological replicates using the ∆∆*C*_t_ method and expression of *tuf* for normalization.

### LC-MS^E^-based protein identification

Bacteria, either containing an empty vector or psLLM2- plasmid (five biological replicates each), were grown in liquid GM17 medium supplemented with 5 µg/ml of chloramphenicol until the desired growth phase. The biomass was collected by centrifugation at 1 800 × *g* for 20 min at 4°C. 20 OD units of bacteria were suspended in 1 ml of freshly prepared urea buffer (8 M urea in 0.1 M Tris–HCl, pH 8.5), homogenized twice for 40 s at 5 m/s using an MP Biomedicals homogenizer, and centrifuged at 15 000 × *g* for 15 min at 4°C. 100 μg of soluble proteins were mixed with DTT (final concentration 0.1 M), incubated for 15 min at 37°C and 300 rpm, and further processed according to FASP protocol as described in [40] starting with the addition of 50 mM iodoacetamide.

LC-MS^E^ analysis was performed as described in [40] with the following modifications: LC-(UD)MS^E^ data were acquired with mass scan time set to 0.9 s, raw data were processed with the Progenesis QI for proteomics version 2.0.5 (Nonlinear Dynamics; Waters Corporation); and the UniProt *Lactococcus lactis* subsp. *cremoris* (strain MG1363) database, downloaded on 27 June 2024, was used for protein identification. Label-free quantification of proteins was performed using the unique peptide areas calculated by the Progenesis algorithm. The Limma package was used to perform differential expression analysis. Enrichment analysis was performed using clusterProfiler package [38] against KEGG database [39].

### Construction and verification of Δ*sLLM1042*+ strain

For the sRNA deletion, the modified pLCNICK was used. Briefly, the parent plasmid was digested with XbaI and Bsp1407I and blunt-ligated with fragment containing P32 promoter amplified from pVE-P32 plasmid with pVE_seq_primerF/pVE_seq_primerR primer pair resulting in plasmid pLCNICK_P32. Then, upstream and downstream regions of sLLM1042+ were amplified from *L. cremoris* gDNA with primer pairs 1042Cs9upF/1042Cs9upR and 1042Cs9downF/1042Cs9downR. The sgRNA-encoding fragment was amplified from pLCNICK with primer pair 1042sgRNA/gTEMdnMOD. These fragments were joined by overlap extension PCR using Phusion polymerase (Thermo Scientific) and primer pair 1042sgRNA/1042Cs9downR, and blunt-ligated into pLCNICK_P32 pre-digested with Bsp1407I and Bsp120I. This plasmid was electroporated into *L. cremoris*, and transformants were selected on GM17 medium with 10 µg/ml erythromycin. Deletion was confirmed by amplifying the region with primer pair R_LLM_1042_PaeI/1042checkF. The deletion plasmid was removed by iteratively plating clones onto non-selective media until they lost the resistance marker. The mutant was confirmed by sequencing amplified regions, performing RT-qPCR, and Northern blot analysis.

pVE-P32 plasmid was generated by introducing the P32 promoter sequence from [41] by invert amplifying and blunt ligating pVE3916 plasmid with primer pair F_invert_pVE_P32/R_invert_pVE_P32. All oligonucleotides used are listed in Supplementary Table S5.

For RT-qPCR analysis, overnight cultures of *L. cremoris* with pMSP3545, *L. cremoris* Δ*sLLM1042*+ with pMSP3545, or pNis-sLLM1042+ were diluted 1:50 and grown in liquid GM17 medium supplemented with 10 µg/ml erythromycin to mid-exponential phase (OD_600_ 0.5–0.8), induced with 5 ng/ml nisin, and collected by centrifugation. One milliliter of biomass per strain was used for RNA extraction using 1 ml TRIzol reagent (Fisher Scientific). The mixture was added to 2 ml homogenization vials containing 250 µl of 0.1 mm zirconia-silica beads and 200 µl of chloroform. Bacteria were homogenized twice for 45 s at 4.5 m/s using MP Biomedicals homogenizer. Vials were centrifuged at 10 000 × *g* for 10 min at 4°C. Total RNA from the supernatants was extracted according to the manufacturer’s recommendations. The integrity of RNA was confirmed using agarose gel electrophoresis. RT-qPCR was performed as described earlier.

For Northern blot analysis, *L. cremoris* with pMSP3545 and *L. cremoris* Δ*sLLM1042*+ with pMSP3545, pNis-sLLM1042+, or pNis-2×MS2-sLLM1042+ were grown overnight in liquid GM17 medium supplemented with the appropriate antibiotic. The following day, cultures were diluted 1:50 into 40 ml of fresh medium and grown until an OD_600_ of 0.7–1 was reached. Expression was induced by adding 0.1 ng/ml nisin and incubating for 15 min. Total RNA was extracted using Trizol reagent, as described earlier. RNA integrity was assessed via gel electrophoresis. Approximately 2 µg of RNA was fractionated using denaturing acrylamide gel electrophoresis and transferred onto a Nucleobond membrane (Macherey-Nagel), which was cross-linked with UV light for 1 min 45 s at 25 J (UVITEC). Subsequent steps were performed as described previously [16]. The oligonucleotides used are listed in Supplementary Table S4.

### MAPS experiment

GST-2×MCP was purified following overnight induction with 0.1 mM Isopropyl-β-d-thiogalactopyranoside (IPTG) at 16°C. Cells were harvested by centrifugation at 5 000 × *g* for 10 min at 4°C. The pellet was resuspended in buffer A (20 mM HEPES, pH 7.9, 100 mM KCl, 1 mM DTT, 1 mM ethylenediaminetetraacetic acid (EDTA), 0.25 mM phenylmethylsulfonyl fluoride (PMSF). Cell lysis was performed using a SONICS Vibra-Cell™ ultrasonic processor (2 × 5 min, 30% amplitude, 3 s on/off cycles), and lysates were clarified by centrifugation at 10 000 × *g* for 15 min at 4°C. The supernatant was applied to a GSTrap HP column (Cytiva). Bound GST-2×MCP fusion protein was eluted using buffer B (20 mM HEPES, pH 7.9, 50 mM KCl, 1 mM DTT, 1 mM EDTA, 20 mM reduced glutathione). Peak fractions were pooled and further purified on a HiPrep Heparin FF 5 ml column (Cytiva). Elution was carried out using a 0%–100% linear gradient of buffer C (20 mM HEPES, pH 7.9, 500 mM KCl, 1 mM DTT, 1 mM EDTA).

The MAPS assay was performed as described by [42], with extensive modifications as detailed below. *L. cremoris* Δ*sLLM1042*+ strains harbouring either pNis-2×MS2-sLLM1042+ or pNis-sLLM1042+ were grown overnight, diluted 1:50 in fresh medium, and cultured to an OD_600_ of 0.8. Expression of sRNA was induced with 5 ng/ml nisin for 15 min. 25 ml of cells were harvested by centrifugation at 5 000 × *g* for 10 min at 4°C, resuspended in 2 ml of 1 × phosphate buffered saline (PBS) supplemented with 1 mM DTT and 0.4 mM PMSF, and homogenized twice for 40 s at 5 m/s using MP Biomedicals homogenizer. Lysates were clarified by centrifugation at 15 000 × *g* for 15 min at 4°C.

Glutathione Sepharose 4B resin (Fisher Scientific) was prepared according to the manufacturer’s recommendations. 25 µl of the slurry per sample was pelleted at 500 × *g* for 5 min and washed three times with 1 × PBS containing 1 mM DTT and 0.4 mM PMSF. 10 µg (25 μl) of purified GST-2×MCP protein was added and incubated for 10 min at 4°C with rotation. The resin was washed twice with 0.5 ml of the same buffer. The cleared lysate was applied to the resin, followed by four washes with 0.5 ml of the same buffer. RNA was eluted in 1 × PBS containing 1 mM DTT and 10 mM reduced glutathione. An equal volume of Roti-Aqua-PCI solution (Carl Roth) was added, mixed, and centrifuged at 13 000 × *g* for 5 min at 4°C. RNA was precipitated by adding three volumes of 99.9% ethanol, followed by centrifugation at 13 000 × *g* for 40 min at 4°C. Pellets were washed twice with 70% ethanol and centrifuged at 13 000 × *g* for 10 min at 4°C. RNA concentration and purity were measured using a NanoDrop 2000 spectrophotometer.

cDNA libraries were prepared, including DNase treatment and rRNA removal, and deep, bidirectional sequencing was performed at Novagene using the NovaSeq X Plus Series (PE150).

Raw sequencing reads were trimmed using trimGalore! [43] and mapped using hisat2 [44]. Gene expression was evaluated using StringTie [36]. To identify potential targets, we used DESeq2 [45]. IntaRNA [46] was used to predict RNA–RNA interactions.

### GFP reporter assay

pVE-P32-glpF3-GFP+ was co-transformed into the Δ*sLLM1042*+ strain with either an empty vector pMSP3545 or a plasmid expressing sLLM1042+ or sLLM1042+m[30–33] from a nisin-inducible promoter. Cultures were grown overnight with 1 ng/ml nisin, diluted to an OD_600_ of 0.1, and sRNA expression was further induced with 10 ng/ml nisin. At an OD_600_ 1.0, cells were collected at 4000 × *g*, washed once, and resuspended in PBS to an OD_600_ 5. GFP+ fluorescence was measured in black plates using BioTek Synergy H4 plate reader (excitation 475 nm; emission 510 nm). Fluorescence values were normalized to the empty-vector control, which was set to 1.

## Results

### Genome-wide identification of *Lactococcus cremoris* sRNAs responding to penicillin G and lysozyme treatment

**Figure 1.**
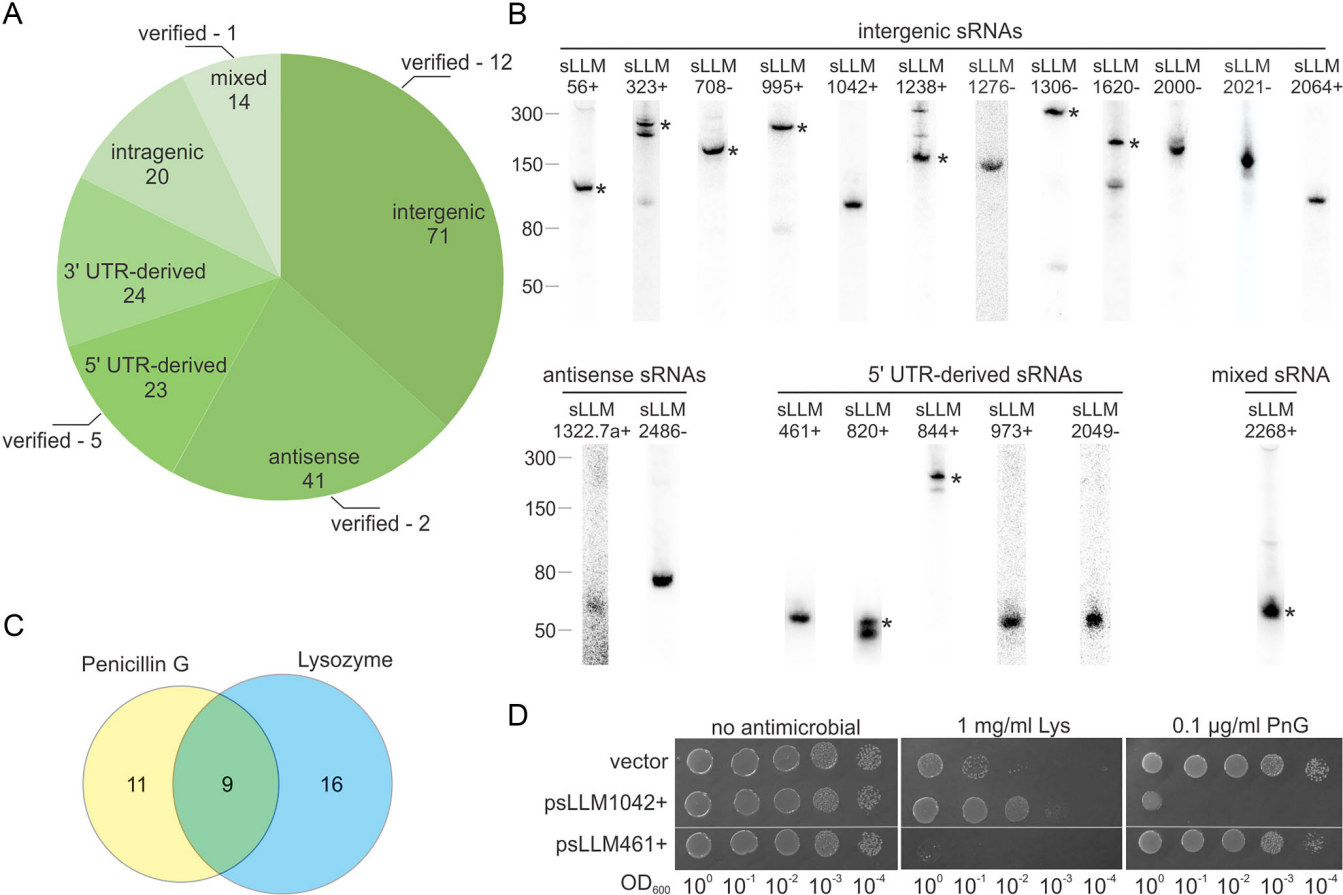
Analysis of sRNAs expressed in *L. cremoris* MG1363 during antimicrobial treatment. (**A**) Group distribution of putative sRNAs in unaffected cells and those treated with penicillin G (PnG) and lysozyme (Lys) under exponential growth and in a cDNA library prepared from cells collected at five growth points. Mixed–sRNAs that can be assigned to more than one category. (**B**) Northern blot validation of 20 selected sRNAs predicted by RNA-seq. If more than one band is visible, an asterisk indicates the sRNA corresponding to the length of the most abundant form, as determined by RNA-seq read coverage. (**C**) Venn diagram of differentially expressed sRNAs after treatment with penicillin G or lysozyme. (**D**) The impact of elevated levels of sLLM1042+ and sLLM461+ on bacterial susceptibility to Lys and PnG. Serial dilutions of the MG1363 culture carrying an empty pVE3916 or an sRNA-containing plasmid were grown on GM17 medium without and with antimicrobial. The results provided are representative of three biological replicates.

To decipher the response of *L. cremoris* MG1363 to two major cell wall-targeting antimicrobials, penicillin G (PnG) and lysozyme, sRNA sequencing was performed. Selected concentrations of penicillin G and lysozyme, 0.125 µg/ml and 0.5 mg/ml, respectively, did not induce bacterial death but already had a minor effect on the growth rate and maximum OD reached (Supplementary Fig. S1A). At OD_600_ 0.5, these stressors were added to the bacterial growth medium, and bacteria were collected after reaching the mid-exponential phase (OD_600_ 1.6–1.8), ~70–80 min later. RNA of 50–500 nt in length was purified, and cDNA libraries were sequenced for the unaffected cells and those treated with PnG and lysozyme. Knowing that the expression of some sRNA species depends on the growth stage, to gain a comprehensive understanding of the total sRNA landscape in *L. cremoris*, we also performed the sequencing of small RNAs purified from bacteria collected at five growth points from lag to late stationary phase (Supplementary Fig. S1B). To minimize false positives and assign as many true sRNAs as possible, they had to satisfy three strict criteria: (i) exhibit a sharp drop in coverage at the 3′ and 5′ ends, (ii) have more than five counts per million per library, and (iii) be present in more than two replicates of whichever condition. By combining the results from all libraries, we determined that, under our experimental conditions, a total of 193 putative unique sRNAs were expressed, comprising 71 intergenic, 41 antisense, 23 and 24 derived from 5′ or 3′ untranslated regions, respectively, 20 intragenic, and 14 categorized as mixed sRNAs—they can be assigned to more than one category (Fig. 1A, Supplementary Fig. S2, and Tables T1–T8 in Supplementary Dataset 1). Of these, 62 corresponded to previously identified sRNA species [47], while the remaining 131 were newly identified (Table T9 in Supplementary Dataset 1). Northern blot analysis confirmed the expression of 20 sRNAs—10 newly characterized and 10 previously reported—thereby validating the accuracy of the sequencing-based predictions in both datasets (Fig. 1B).

Differential analysis revealed significant changes in the expression of 36 sRNAs following antimicrobial treatment (Table 1 and Table T10 in Supplementary Dataset 1). Specifically, the expression of 20 sRNAs was altered after exposure to PnG, with 16 downregulated and 4 upregulated, while 25 sRNAs were affected by lysozyme treatment, with 23 downregulated and 2 upregulated. Notably, a quarter of these sRNAs responded to exposure to each of the tested antimicrobials (Fig. 1C). To evaluate the impact of antimicrobial-responsive sRNAs on bacterial resistance, two representatives were selected from the 36 differentially expressed sRNAs: sLLM461+, which was induced 1.8-fold exclusively by penicillin G, and sLLM1042+, which was repressed under both lysozyme (3.1-fold) and penicillin G (2.0-fold) treatments. These sRNAs, previously validated by Northern blot, were cloned into the pVE3916 vector along with their surrounding regions, including promoter sequences. After sRNA overexpression was confirmed by Northern blot (Supplementary Fig. S3), overnight cultures of plasmid-containing bacteria were spotted onto GM17 agar supplemented with either 1 mg/ml lysozyme or 0.1 µg/ml PnG (Fig. 1D). Interestingly, while the expression of intergenic sLLM1042+ decreased 2–3-fold after exposure to both PnG and lysozyme, its overexpression resulted in the opposite effect on the bacteria’s response to these antimicrobials. Specifically, sensitivity to PnG was markedly enhanced, whereas sensitivity to lysozyme was decreased. Meanwhile, the overexpression of sLLM461+ increased bacterial susceptibility to lysozyme but had no impact on resistance to PnG, despite the RNA-seq data showing that the cellular expression of this 5′ UTR-derived sRNA nearly doubled after exposure to PnG. Even though the observed phenotypes reflect the effect of elevated sRNA dosage, at the same time, these findings indicate that the relationship between sRNA expression and antimicrobial response is multifaceted and condition-dependent, rather than following a straightforward correlation. Furthermore, as illustrated by the analysed examples, individual sRNAs can exert opposite effects on resistance to different cell wall-targeting agents, highlighting the complexity of sRNA-mediated regulation.

**Table 1.** List of *L. cremoris* sRNAs differentially expressed after antimicrobial treatment, as determined by RNA-seq

		log_2_FC after treatment with		Validated by Northern blot
sRNA	Group	Lysozyme	Penicillin G	
sLLM56+	Intergenic	**−1.166**	−0.683	+
sLLM87+	Antisense	**−0.870**	−0.667	
sLLM166+	Intragenic	**−1.035**	−0.707	
sLLM323+	Intergenic	**−1.320**	−0.263	+
sLLM461+	5′ UTR-derived	0.188	**0.844**	+
sLLM585+	Antisense	−0.553	**−0.842**	
sLLM820+	5′ UTR-derived	−0.701	**−0.924**	+
sLLM861+	Intergenic	−0.405	**−0.802**	
sLLM873+	Intergenic	**−0.822**	−0.565	
sLLM898+	3′ UTR-derived	−0.613	**−1.433**	
sLLM1038+	Antisense	**−0.946**	−0.234	
sLLM1042+	Intergenic	**−1.609**	**−1.000**	+
sLLM1043+	5′ UTR-derived	−0.409	**−1.042**	
sLLM1065+	5′ UTR-derived	**−1.139**	−0.102	
sLLM1209−	Intergenic	**−0.967**	−0.116	
sLLM1210+	Intergenic	−0.272	**−0.970**	
sLLM1211−	5′ UTR-derived	**−1.155**	−0.118	
sLLM1228.2+	Intergenic	−0.630	**−0.864**	
sLLM1238+	Intergenic	**1.038**	−0.632	+
sLLM1259−	5′ UTR-derived	**−2.184**	**−1.439**	
sLLM1276−	Intergenic	**−1.442**	**−1.360**	+
sLLM1541−	3′ UTR-derived/antisense	0.792	**1.075**	
sLLM1692−	3′ UTR-derived	**1.150**	**1.159**	
sLLM1729+	Intergenic	**−0.893**	**−0.830**	
sLLM1848−	Intragenic	**−1.043**	−0.486	
sLLM1959+	Antisense	**−1.007**	−0.041	
sLLM2064−	Intergenic	**−0.905**	**−1.368**	
sLLM2064+	Intergenic	**−1.396**	**−1.056**	+
sLLM2065.3−	Intergenic	**−1.567**	**−1.106**	
sLLM2255.1−	Intergenic	**−0.996**	−0.405	
sLLM2258−	Intergenic	**−1.522**	**−1.130**	
sLLM2311−	3′ UTR-derived	−0.511	**−1.317**	
sLLM2332−	Intergenic	**−0.839**	−0.796	
sLLM2343−	3′ UTR-derived	**−0.891**	−0.468	
sLLM2386−	3′ UTR-derived	Not significant	**2.602**	
sLLM2399−	5′ UTR-derived	**−1.109**	0.408	

Altered expressions (log_2_ fold change >0.8, false-discovery rate ≤ 0.1) are indicated in bold.

### Functional screening of *L. cremoris* genome fragments increasing tolerance to antimicrobials targeting the cell wall integrity

**Figure 2.**
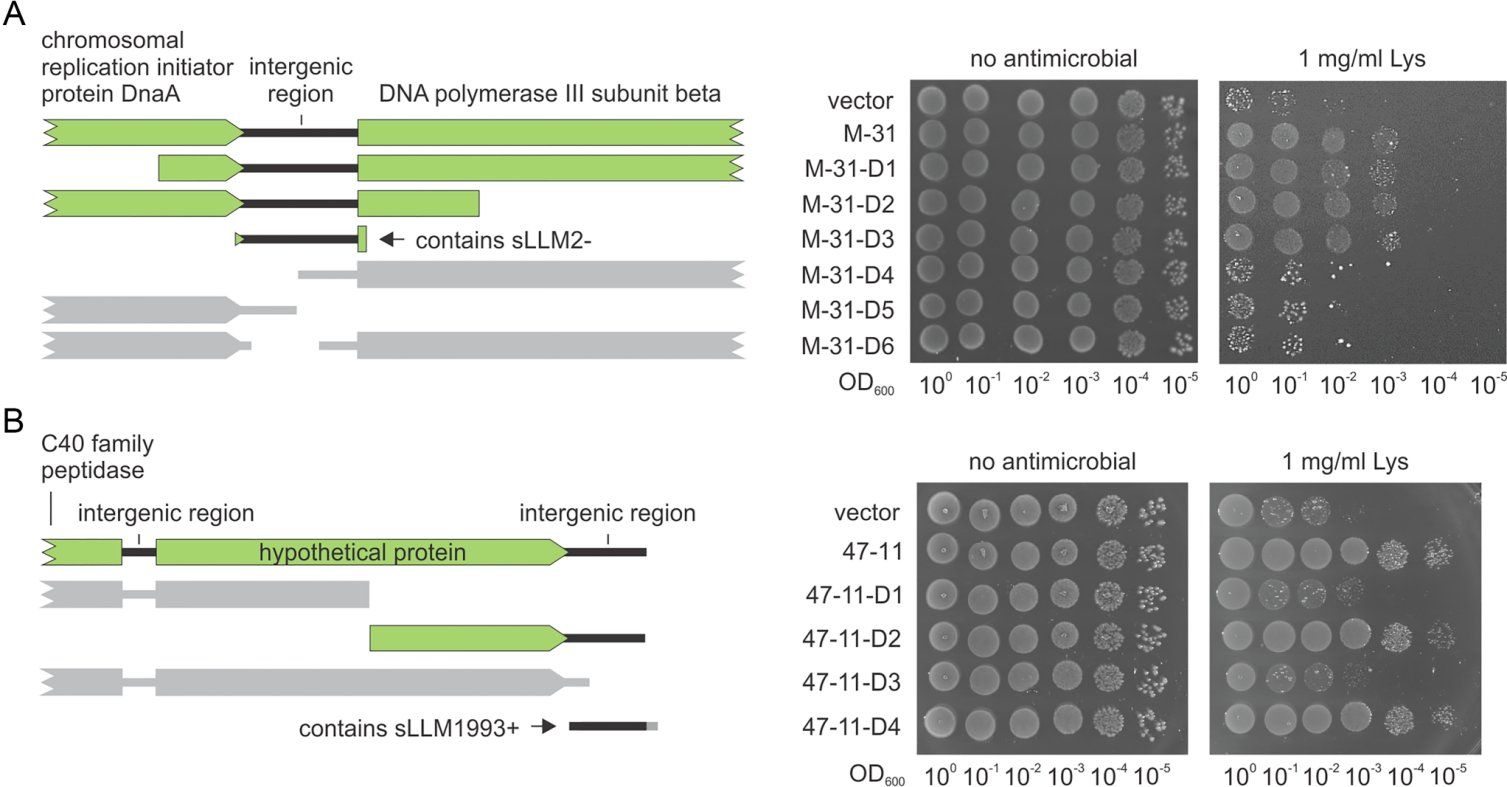
Functional analysis of *L. cremoris* genome fragments altering susceptibility to lysozyme. (**A**) The deletional analysis of the M-31 genomic fragment demonstrated that the intergenic region increases lysozyme (Lys) resistance. Non-functional variants are shown in grey. (**B**) The second intergenic region of the 47–11 genomic fragment is required for lysozyme resistance. In the case of D4, 16 bases were added to the 3′ end of the second intergenic region. The results provided are representative of three biological replicates.

Although transcriptional changes can highlight candidate stress-responsive sRNAs, altered expression does not necessarily imply a direct role in antimicrobial resistance and may not lead to observable phenotypic changes. Therefore, to further identify functionally relevant stress-responsive sRNAs, a classical selection strategy was implemented to expand the list of candidates with antimicrobial activity in *L. cremoris*. To limit the number of full-length protein-coding genes, a gene bank of *L. cremoris* gDNA was constructed, comprising ~25 000 clones with relatively short fragments (average insert size of 410 bp) that collectively cover the whole genome about four times. This plasmid bank was transformed into the WT and Δ*oatA* strains. The latter contains an inactivated PG O-acetyltransferase, OatA, which results in increased sensitivity to lysozyme [48]. After two rounds of selection, two clones of WT strain and four of Δ*oatA* strain that grew at 4 or 2 mg/ml of lysozyme, respectively, were chosen for further analysis (Supplementary Fig. S4). Sequencing of their inserts revealed that four of them (two from each strain) contained identical 1001 bp sequence, covering the genomic fragment from 1075 to 2075 bp (NCBI genomic coordinates: NC_009004.1) and including the end fragment of chromosomal replication initiator protein DnaA, about half of DNA polymerase III subunit beta, and a 156 bp long intergenic region between them (Fig. 2A). Meanwhile, two other inserts selected using the Δ*oatA* strain had an identical sequence of 859 bp, containing a genomic fragment from 1 992 114 to 1 992 972 bp, which included an entire hypothetical protein, the 5′ end of the C40 family peptidase, and two intergenic regions of 48 and 110 bp in length (Fig. 2B). The repeated identification of the same sequences further suggested their role in altering *L. cremoris* susceptibility to lysozyme.

Deletion analysis was conducted to determine the specific regions of the selected genomic fragments responsible for the observed effect. It revealed that the intergenic region of the M-31 fragment in clone D3, spanning genomic coordinates between 1349 and 1537 bp, was a crucial determinant of bacterial resistance to lysozyme (Fig. 2A). Deleting most of this intergenic region, from 1374 to 1468 bp in clone D6, completely abolished the observed phenotype, as indicated by the spotting test. Likewise, the intergenic region in fragment 47-11, spanning between 1 992 863 and 1 992 988 bp, was essential for maintaining *L. cremoris* resistance to lysozyme (Fig. 2B). Inspection of the sequencing data revealed the presence of RNA reads in both intergenic regions, albeit in relatively limited numbers (Supplementary Fig. S5A and B). This suggests that the M-31 and 47-11 fragments may encode small RNAs, which did not meet the criteria for sRNA identification used in our RNA-seq analysis. Indeed, the Northern blot confirmed the presence of both short RNAs, named sLLM2- and sLLM1993+, in *L. cremoris* cells carrying plasmids with an extra copy of the intergenic region (Supplementary Fig. S5C and D). Hereafter, the M-31-D3 and 47-11-D4 constructs are referred to as psLLM2- and psLLM1993+, respectively. Interestingly, expression tended to increase in both cases during the later growth stages. Since they were driven by native promoters, this indicates the existence of specific cellular factors that can regulate transcription or stability of these RNAs.

**Figure 3.**
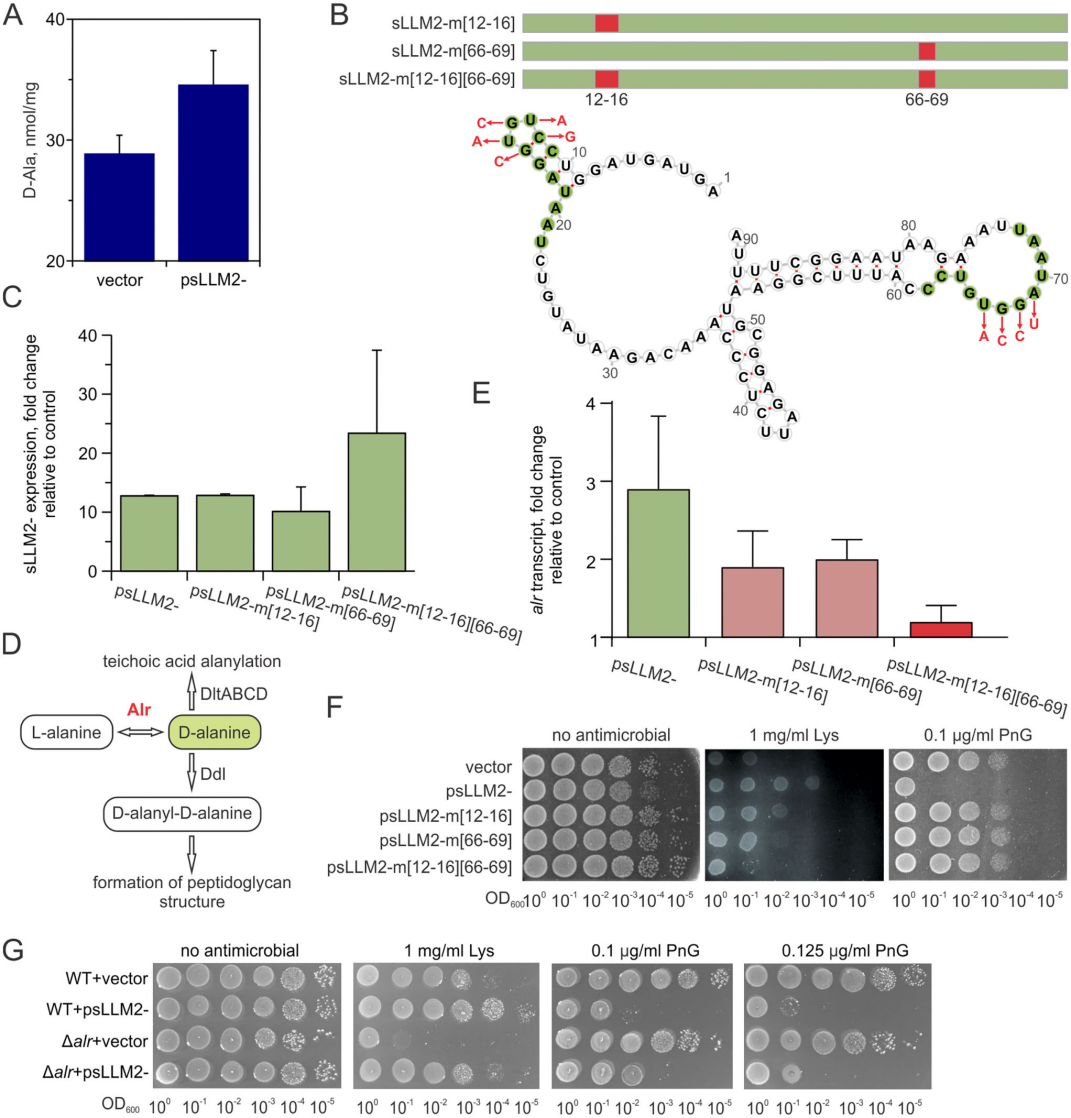
Characterization of the sLLM2- small RNA roles in biological processes. (**A**) High-performance liquid chromatography analysis revealed increased D-alanylation of teichoic acids in the strain expressing sLLM2-. The mean ± SD was calculated from three biological replicates. The Welch *t*-test with pairwise comparisons revealed a significance of *P* < 0.05. (**B**) sLLM2- centroid secondary structure predicted by RNAfold, with direct 12-nucleotide repeats at positions 11–22 and 62–73 nt highlighted with green circles. The mutated nucleotides are shown in red. (**C**) RT-qPCR revealed that intact and mutant variants of sLLM2- are expressed at similar levels. Total RNA from a strain carrying an empty vector was used as a control. The mean ± SD was calculated from three biological replicates. (**D**) Cellular processes affected by alanine racemase Alr. (**E, F**) Mutations at positions 12–16 and 66–69 impair the function of sLLM2- sRNA. At the top (**E**), RT-qPCR analysis confirmed that the double mutant sLLM2-m[12–16][66–69] completely reverted the *alr* expression to the WT level. Total RNA from a strain carrying an empty vector was used as a control. The experiments were performed using total RNA extracted from the mid-exponential growth phase. The results provided were calculated from three biological replicates. At the bottom (**F**), mutations abolished the increased resistance to lysozyme (Lys) and sensitivity to penicillin G (PnG). (**G**) Effect of genomic deletion of *alr* and elevated levels of sLLM2- on bacterial susceptibility to PnG and Lys. Serial dilutions of *L. cremoris* NZ9000 (WT) or NZ3900 (Δ*alr*), a strain carrying a deletion of the alanine racemase gene, transformed with either an empty pVEA1 vector or an sLLM2- containing plasmid, were grown on GM17 agar supplemented with 2 mM D-alanine, with and without antimicrobial compounds. The results provided are representative of two biological replicates.

*L. cremoris* MG1363 transformed with psLLM2- and psLLM1993+ plasmids, containing the intergenic regions, also facilitated bacterial growth in the liquid GM17 medium with 1 mg/ml lysozyme (Supplementary Fig. S6A). Moreover, these plasmids conferred resistance to 0.2 µg/ml of antimicrobial peptide nisin (Supplementary Fig. S6A), which traverses the cell wall and interacts with lipid II in the cytoplasmic membrane, thereby inhibiting PG synthesis and forming pores that ultimately lead to cell death [49]. Still, sLLM2- and sLLM1993+ did not increase the bacterial resistance to lysozyme-derived peptide RAWVAWRNR (data not shown), which, similarly to nisin, is thought to induce the formation of pores due to its hydrophobic and cationic properties through the interaction with the bacterial membrane [7]. Additionally, no increased resistance was observed against vancomycin (data not shown), an antibiotic inhibiting peptidoglycan synthesis through the interaction with the terminal D-Ala-D-Ala groups of un-crosslinked lipid II, thereby disrupting the normal formation of peptidoglycan [50]. On the other hand, the expression of the sLLM2- augmented bacterial sensitivity to penicillin G (Fig. 3F) and polymyxin B, as well as a range of physical factors, such as NaOH, HCl, H_2_O_2_, and NaCl, suggesting that the regulatory functions of this sRNA may extend beyond cell wall-targeting antimicrobials (Supplementary Fig. S6B).

In conclusion, RNA-seq and functional analysis allowed the identification of four small RNAs whose elevated levels altered susceptibility to cell wall-targeting antimicrobials. Of these, sLLM2- and sLLM1042+ were selected for further investigation: sLLM2- was identified in multiple clones selected through functional screening and contributed to high lysozyme tolerance, whereas sLLM1042+ showed expression and phenotypic changes associated with both penicillin G and lysozyme responses, consistent with the primary focus of this study.

### Regulatory effects of sLLM2- sRNA

Morphological analysis revealed no difference between strains containing the pVE3916 vector and the psLLM2- plasmid expressing sLLM2- (Supplementary Fig. S7A). Therefore, to search for a potential regulatory pathway for the selected sRNA, we expressed plasmid-localized sLLM2- in strains with loss-of-function mutations in key genes involved in cell wall biogenesis: Δ*acmA*, Δ*oatA*, Δ*pgdA*, and in a *dltD* mutant. While an increasing sLLM2- levels continued to provide higher resistance to lysozyme in the first three strains, this effect was abolished in the context of *dltD* (Supplementary Fig. S7B). The membrane protein DltD is involved in transferring D-alanine from a carrier protein inside the cell to teichoic acids on the cell surface, suggesting that the effect of sLLM2- may be related to alterations in cellular D-alanine concentration or regulation of the pathway for D-alanylation of teichoic acids. Notably, it has been previously reported that increased levels of D-alanylation led to augmented resistance to both lysozyme and nisin in lactococci [35]. Indeed, while the analysis of peptidoglycan in the sLLM2- expressing strain with the psLLM2- plasmid showed no difference compared to the strain bearing the control vector (data not shown), the D-alanylation of teichoic acids increased from 28.9 ± 1.5 nmol/mg to 34.6 ± 2.8 nmol/mg (Fig. 3A). Galactose is an additional factor that impacts glycosylation—the second type of modification—which, in turn, determines the composition and functionality of cell wall lipoteichoic acids in lactococci [51]. An examination of growth in a liquid M17 medium supplemented with different carbon sources revealed that elevated sLLM2- expression impaired bacterial growth with galactose supplementation but not with glucose or cellobiose (Supplementary Fig. S8A). The susceptibility of *L. cremoris* cells to lysozyme and their resistance to penicillin G appeared to be slightly increased, as examined on the galactose agar medium. Nevertheless, the sLLM2- expressing strain exhibited enhanced lysozyme resistance and susceptibility to penicillin G on both glucose and galactose media (Supplementary Fig. S8B), suggesting that these functions of the examined sRNA are not affected by growth in the presence of different carbohydrates and likely the extent of teichoic acid galactosylation.

**Figure 4.**
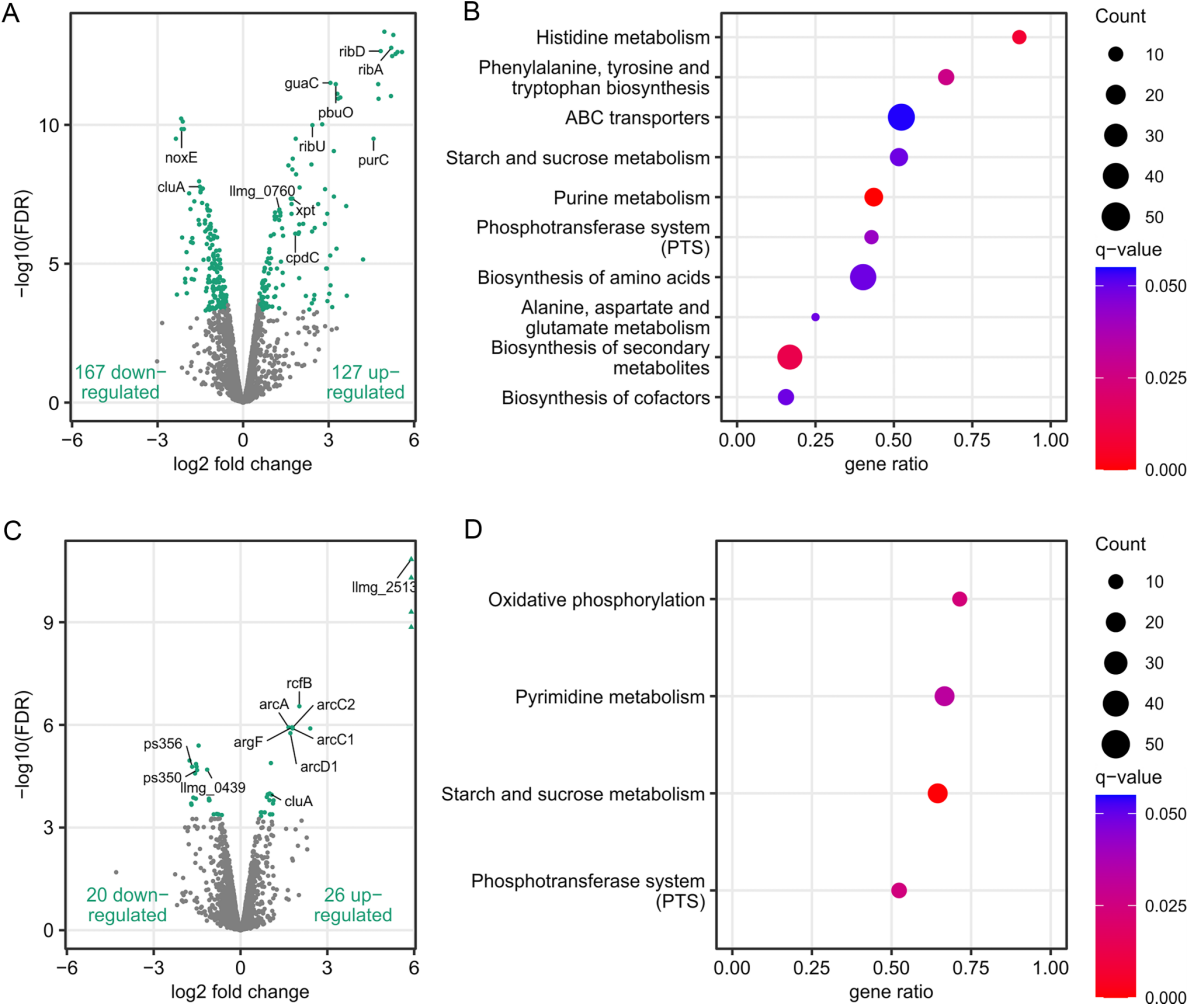
Transcriptomic analysis of changes in the strain with enhanced sLLM2- or sLLM1042+ expression. (**A**, **C**) The Volcano plot of differentially expressed transcripts following increased sLLM2- and sLLM1042+ expression, respectively. Significantly dysregulated RNAs that passed the >1.5-fold change and < 0.0005 FDR thresholds are shown in green. Transcripts with more than sixfold upregulation, protruding beyond the axis limit, are indicated by triangles. Transcripts of genes additionally analysed by RT-qPCR are also indicated by their gene names. (**B, D**) Gene set enrichment analysis (GSEA) of the differentially expressed transcripts.

To better understand the impact of increased sLLM2- expression on the observed phenotype, we performed transcriptomic and proteomic analyses. Whole transcriptome cDNA libraries were prepared from bacteria grown until the mid-exponential phase, carrying either the empty vector or the psLLM2- plasmid (Supplementary Fig. S1C). Differential RNA-seq analysis revealed 294 variable genes (127 upregulated and 167 downregulated) after sLLM2- overexpression [FC > 1.5, false discovery rate (FDR) < 0.0005], with changes in the expression of genes related to metabolic pathways, especially amino acids, purine, and sugar metabolism, biosynthesis of cofactors and secondary metabolites, ABC transporters, and phosphotransferase system (Fig. 4A and B and Table T11 in Supplementary Dataset 2). RT-qPCR analysis confirmed the reliability of the RNA-seq data for 12 mRNAs, including five associated with purine metabolism and three with riboflavin biosynthesis, as well as mRNAs encoding NADH oxidase, a surface protein involved in cell aggregation, and a putative transglycosylase (Supplementary Table S8).

**Figure 5.**
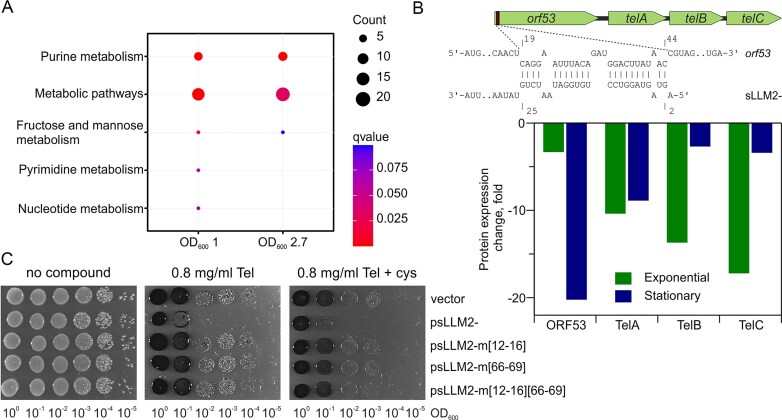
Elevated levels of sLLM2- are associated with increased sensitivity to tellurite. (**A**) KEGG pathway overrepresentation analysis of significantly altered proteins after increased sLLM2- expression during exponential and stationary phases. (**B**) At the top, the scheme of predicted sLLM2- interactions with the presumed operon consisting of the putative tellurium resistance proteins ORF53, TelA, TelB, and TelC, corresponding to A2RKX4, A2RKX5, A2RKX6, and A2RKX7 in Tables T12 and T13 in Supplementary Dataset 3, respectively. At the bottom, the effect of increased sLLM2- expression on the protein abundance in exponential and stationary phases, compared to the control strain harbouring the plasmid without sRNA, based on proteomic analysis. (**C**) Growth of strains over 24 h with enhanced expression of sLLM2- or its mutant in the presence of potassium tellurite (Tel) with or without cysteine. The black colour results from the formation of metallic Te^0^.

Proteomic analysis was performed on two sets of samples collected at the mid-exponential (OD_600_ 1.0) and stationary (OD_600_ 2.7) growth phases. The changes in 44 and 63 (fold change ≥ 1.5, adjusted *P*-value < 0.05) protein levels were detected in strains containing the plasmid with sLLM2- sRNA at earlier and later bacterial growth stages, respectively (Tables T12 and T13 in Supplementary Dataset 3). Proteomic dataset enrichment analysis corroborated the transcriptomic results, confirming that sLLM2- expression may affect metabolic pathways, particularly nucleotide (purine) and sugar metabolism (Fig. 5A). The most notable decrease was observed in the expression of four putative tellurium resistance proteins, A2RKX4–A2RKX7 (Fig. 5B). However, the transcriptomic data showed no significant changes in RNA abundance (*LLMG_RS06855*-*LLMG_RS06840*), which may belong to the same operon (Table T11 in Supplementary Dataset 2), indicating that regulation occurs at the level of protein synthesis. Consistent with this observation, rifampicin-based RNA stability assays (following inhibition of transcription initiation) in bacteria harbouring an empty vector control or expressing sLLM2- revealed no effect of sLLM2- sRNA on *orf53* stability (data not shown). This is in line with previous findings in Gram-positive bacteria, where numerous sRNAs are known to modulate gene expression primarily at the translational rather than transcriptional level [52–56]. To confirm the bioinformatically predicted functions of these proteins, we grew the bacteria in a medium supplemented with potassium tellurite. Indeed, the native sLLM2- expressing strain revealed increased sensitivity to this toxic compound alone and in combination with cysteine (Fig. 5C).

The sequence of sLLM2- appeared peculiar in the way that it carried direct 12 nt repeats (5′-CCTGTGGATAAT-3′) at positions 11–22 and 62–73 nt, both of which covered the hairpin loop structures conserved between *L. cremoris* strains (Supplementary Fig. S9). Hypothesizing that these elements might be essential for sRNA interaction with mRNA or protein targets, we constructed three mutants: two with a single modified element, sLLM2-m[12–16] (CUGUG was changed to GACAC) and sLLM2-m[66–69] (UGGA was changed to ACCU), and a double mutant with both regions modified, sLLM2-m[12–16][66–69] (Fig. 3B). Notably, IntaRNA predicted that the first direct repeat region (positions 11–22) base-pairs with *orf53*—the first gene in an operon encoding putative tellurium resistance proteins—and with *alr*, involving the interactions mediated by the mutated nucleotides at positions 12–16 and 13–15, respectively (Fig. 5B and Supplementary Fig. S10). The expression levels of native and modified sRNA variants were comparable, indicating that the mutations had no significant effect on transcription efficiency or stability (Fig. 3C). Mutations in sLLM2- restored the abundance of tellurium resistance proteins (data not shown) and re-established increased tolerance to the oxyanion tellurite (Fig. 5C). These data indicate that an intact, fully functional sLLM2- is required for the observed effect. Moreover, to our knowledge, these results provide the first functional link between the *orf53, telA, telB, telC* locus and tellurite susceptibility in *L. cremoris*.

While *dltD* initially appeared as a plausible direct target of sLLM2-, neither *dltD* transcript nor DltD protein levels showed any change upon sLLM2- overexpression. Similarly, other *dlt* genes were not transcriptionally affected, and their protein products were either unchanged or undetected (Table T11 in Supplementary Dataset 2, Tables T12 and T13 in Supplementary Dataset 3). Instead, the increased expression of the *alr* gene, both at the transcript (increased by 1.60-fold) and protein level (by 1.94-fold at the stationary phase), which encodes alanine racemase interconverting L-alanine to D-alanine, may provide a link between sLLM2- and teichoic acid D-alanylation. Elevating cellular D-alanine levels might influence LTA modification and peptidoglycan synthesis processes, thereby altering lysozyme and penicillin resistance (Fig. 3D). RT-qPCR experiments validated the increase observed in RNA-seq, detecting 2.9 ± 0.9-fold change in *alr* transcription following the elevation of sLLM2- (Fig. 3E, green bar). Notably, the double sLLM2- mutant exhibited a reduction in *alr* RNA to WT levels, while the *alr* mRNA levels in the single element mutants were intermediate between cells containing the empty vector and those with sLLM2- sRNA-expressing plasmid (Fig. 3E, red and pink bars, respectively). To examine what kind of effect these mutations might have on the response to cell wall-affecting factors, we explored the resistance of bacteria expressing intact or mutant sLLM2- to the treatment of lysozyme and penicillin G using a gradient dilution drop plate experiment. As shown in Fig. 3F, only the double mutant completely restored the native sensitivity to lysozyme, while the single-element mutants displayed intermediate phenotypes. This result mirrors the gradient alterations in *alr* transcript levels observed using different mutants, suggesting that lysozyme resistance may be regulated by sLLM2- through modulation of *alr* level. In contrast, all mutations consistently reduced bacterial sensitivity to penicillin G, indicating this response is either less sensitive to *alr* alterations or governed by another mechanism. To directly assess the contribution of *alr* to lysozyme resistance, we examined the response of the Δ*alr* strain to cell wall-acting agents. As shown in Fig. 3G, deletion of *alr* resulted in a marked increase in lysozyme sensitivity, while susceptibility to penicillin G remained unchanged. Notably, overexpression of sLLM2- still enhanced lysozyme tolerance in the Δ*alr* background. Collectively, these findings suggest that the effects of elevated sLLM2- expression on susceptibility to cell wall-targeting antimicrobials involve *alr*-dependent modulation of D-alanine metabolism and parallel regulatory mechanisms.

### Regulatory effects of sLLM1042+ sRNA

For a detailed analysis of the second antimicrobial response-modulating sRNA, sLLM1042+, we explored the effect of its enhanced expression on bacterial morphology and response to a broad range of stressors. The results demonstrated marginal enhancement in chain formation (Supplementary Fig. S11A) and no discernible changes in cell wall structure, as observed by transmission electron microscopy (data not shown). The phenotype associated with enhanced sLLM1042+ expression across a broad range of stressors was quite similar to that observed for sLLM2-, since it likewise increased bacterial resistance to lysozyme and sensitivity to penicillin G (Fig. 1D), NaOH, HCl, H_2_O_2_, polymyxin B, and heat. However, unlike sLLM2-, it did not affect sensitivity to NaCl (Supplementary Fig. S11B).

To delineate transcriptomic alterations occurring in *L. cremoris* following increased expression of sLLM1042+, total RNA extracted from cells harvested at the mid-exponential phase (OD_600_ 0.8) was sequenced (Supplementary Fig. S1C). Differential analysis of RNA-seq data, using FC > 1.5 and FDR < 0.0005 as selection criteria, revealed a total of 46 genes with changed expression, of which 26 were up-regulated and 20 down-regulated (Fig. 4C and Table T14 in Supplementary Dataset 4). RT-qPCR experiments confirmed the altered expression of 11 selected genes, corroborating the validity of the RNA-seq dataset (Supplementary Table S9). The most significant change in expression (282–392-fold) was detected for genes encoding a hypothetical protein, universal stress protein, and MFS transporter (*LLMG_RS12635, LLMG_RS12630, LLMG_RS12625*), which appear to comprise a putative coordinately regulated operon. Previous studies reported that this operon was highly dysregulated after bacteria were exposed to heat, acid, and osmotic conditions for 5 min [57], and that transcription of the MFS transporter (formerly named *llmg_2513* and *yxbD*) was significantly increased in response to acidic stress [58] and in a cholate- and rhodamine-resistant strain [59]. These findings indicate that this change may represent a general stressor response pathway. Meanwhile, GSEA revealed significant enrichment in oxidative phosphorylation, phosphotransferase system, pyrimidine, and starch and sucrose metabolism pathways (Fig. 4D). As in the case of enhanced sLLM2- expression, a portion of the affected genes appeared to be associated with sugar uptake and regulation of metabolism (phosphotransferase system and starch and sucrose metabolism pathways). Indeed, increased expression of sLLM1042+ inhibited bacterial growth in liquid M17 medium supplemented with cellobiose but had very weak, if any, effects in the case of glucose or galactose (Supplementary Fig. S11C).

Searching for differentially expressed RNAs that might be involved in cell wall formation enabled the identification of several genes that may play a role in the antimicrobial stress response. Particularly, the down-regulation of *dacB* transcript (*LLMG_RS07950*), encoding D-alanyl-D-alanine carboxypeptidase family protein, may alter peptidoglycan structure and potentially affect the antimicrobial phenotype. To assess if this gene contributes to the acquired lysozyme resistance after sLLM1042+ expression, we transformed a set of *L. cremoris* mutant strains with the psLLM1042+ plasmid. The persistence of enhanced lysozyme resistance in Δ*dacA* and *dacB* strains (Supplementary Fig. S11D) and the tendency of *dacB* cells to remain associated in chains (Supplementary Fig. S11A) suggest that neither of these genes is essential for mediating the observed effects. In contrast, *pgdA* deletion restored sensitivity to the antimicrobial, indicating that deacetylation of N-acetylglucosamine may interfere with the sRNA activity.

The transcription of another gene (*LLMG_RS04350*), encoding a putative endolysin—protein containing two LysM peptidoglycan-binding domains—was decreased 3.00-fold. LysM domains frequently serve as anchors for autolysins, such as AcmA, to peptidoglycan, thereby contributing to peptidoglycan remodelling [60]. The expression of the neighbouring holin candidate (*LLMG_RS04345*) was also downregulated 2.92-fold, consistent with coordinated repression of the holin-endolysin system. Additionally, a 1.91-fold decrease in expression of FtsX-like permease (a component of the putative FtsEX cell-wall remodelling complex) was determined. Collectively, these changes are consistent with reduced cell wall hydrolysis/autolytic capacity [61] and may be associated with increased lysozyme resistance and longer chain morphology. Taken together, the transcriptomic and phenotypic analyses following sLLM1042+ overexpression suggest a pleiotropic role of this sRNA in modulating bacterial resistance and stress response. However, further studies are required to confirm these proposals.

### Identification of candidate sLLM1042+ interactors using MAPS technology

**Figure 6.**
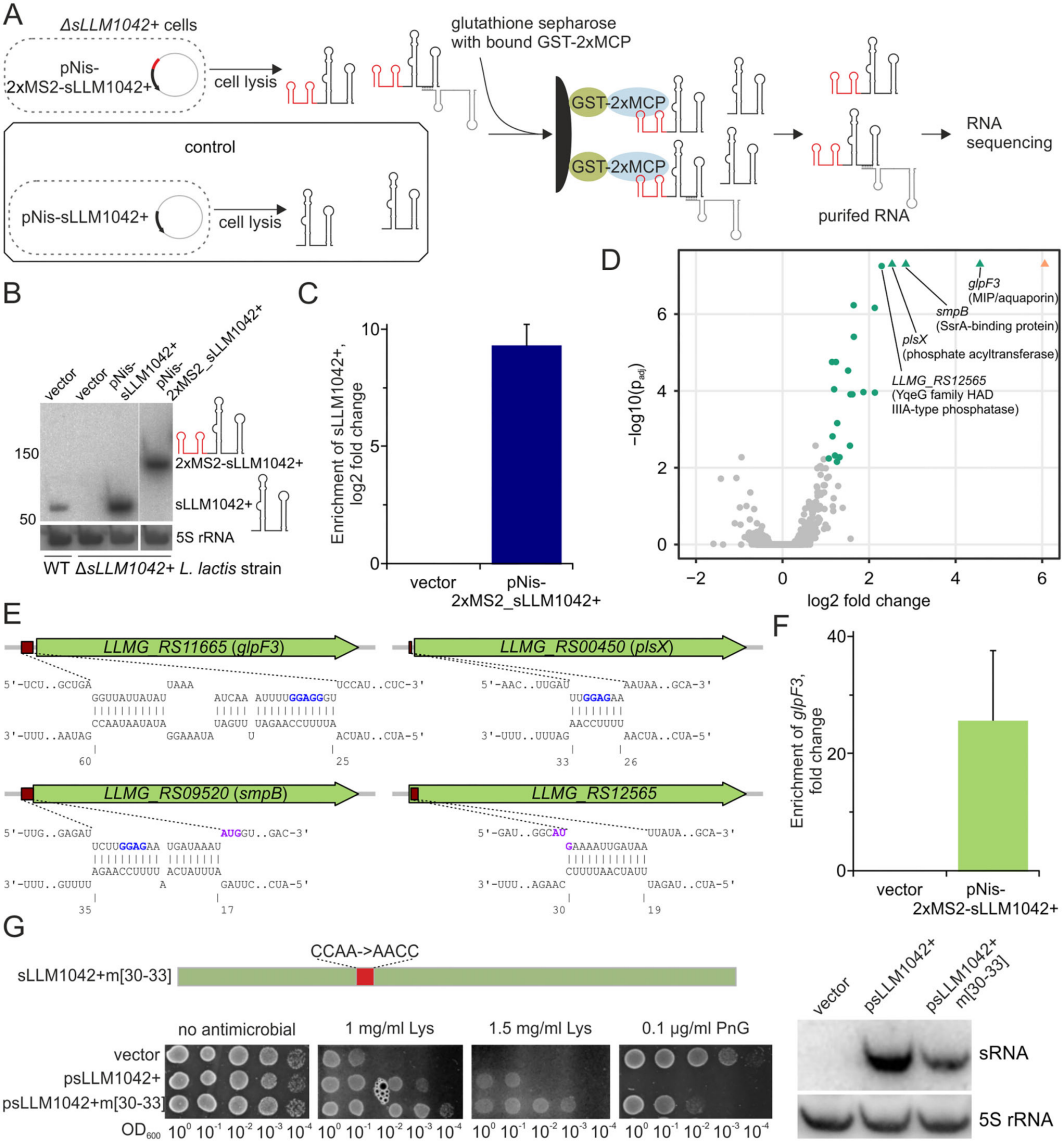
Mapping of candidate sLLM1042+ RNA targets using the MAPS assay. (**A**) Scheme of MAPS experiment. (**B**) Assessment of sLLM1042+ levels in WT *L. cremoris* MG1363 and the Δ*sLLM1042*+ deletion mutant. A double MS2 stem-loop RNA aptamer is fused to the 5′-end of sLLM1042+ in 2×MS2-sLLM1042+. 5S rRNA was used as an internal loading control. (**C**) RT-qPCR confirmed the enrichment of 2×MS2-sLLM1042+ from Δ*sLLM1042*+ strain lysate following affinity purification using GST-tagged 2×MCP. *P*-value < 0.01 indicated a statistically significant result compared to the control. (**D**) Volcano plot of enriched pulled-down transcripts. RNAs that passed the thresholds of fold change >2 and *P* adjusted < 0.001 are highlighted in green. Triangles indicate transcripts with adjusted *P*-values extending beyond the axis limit. (**E**) Predicted sLLM1042+ interactions with the four most enriched mRNA targets. SD boxes are highlighted in blue, and translation START sequences are highlighted in purple. (**F**) RT-qPCR confirmed the RNA-seq-revealed enrichment of *glpF3* from Δ*sLLM1042*+ strain lysate. The mean ± SD was calculated from two biological replicates. (**G**) Characterization of the sLLM1042+m[30–33] mutant with altered sRNA region interacting with the SD box. Left, effect of the 30–33 nucleotide substitutions on sRNA-mediated resistance to lysozyme and sensitivity to penicillin G. Right, Northern blot showing the sRNA expression level.

However, not all transcripts may be directly controlled by sLLM1042+. Moreover, sRNAs can modulate target gene expression through diverse mechanisms, such as base pairing with target RNA or sequestering regulatory proteins. To complement our previous findings and gain deeper insight into potential primary RNA targets of sLLM1042+, we employed an *in vivo* tagging MS2-affinity purification protocol coupled with the RNA sequencing technology (MAPS) (Fig. 6A). To minimize interference from the genomic sRNA, using CRISPR-Cas technology, we constructed a Δ*sLLM1042*+ strain lacking the sLLM1042+ genomic copy (Supplementary Fig. S12). Expression of plasmid-borne sLLM1042+ fused to a double MS2 stem-loop RNA aptamer was shown to be comparable to that of native sLLM1042+ after 15 min of induction with nisin in the Δ*sLLM1042*+ background (Fig. 6B). Following affinity purification using GST-tagged 2×MCP (MS2 coat protein), 2×MS2-sLLM1042+ was enriched nearly 800-fold (log_2_FC = 9.64 ± 0.97) over sLLM1042+, confirming the method’s high selectivity (Fig. 6C). Sequencing of RNA interacting with sLLM1042+, obtained from the MAPS assay, revealed the enrichment of 22 protein-coding RNA regions with log_2_FC > 1 and adjusted *P*-value < 0.001 (Fig. 6D and Table T15 in Supplementary Dataset 5). As nine of them were found to be components of four putative operons (e.g. the neighbouring genes *LLMG_RS11540, LLMG_RS11535*, and *LLMG_RS11530* belonged to the same operon), a total of 17 transcripts interacting with the tested sRNA were identified. Interestingly, only two (*LLMG_RS00975* and *LLMG_RS09015*) exhibited more than a 1.5-fold change in RNA transcript levels, suggesting that sLLM1042+-mediated regulation may primarily occur at the translation level. Indeed, IntaRNA analysis of the four predominantly enriched genes predicted a thermodynamically favourable interaction between sLLM1042+ and the *LLMG_RS11665* (*glpF3*; Δ*G* value of −11.3), *LLMG_RS09520* (*smpB*; Δ*G* value of −12.6), *LLMG_RS00450* (*plsX*; Δ*G* value of −4.7), or *LLMG_RS12565* (Δ*G* value of −7.4) regions in the vicinity of the Shine-Dalgarno box/translation start codon, thereby supporting the possibility of translational inhibition (Fig. 6E). A 25-fold enrichment of *LLMG_RS11665* mRNA, obtained by the RT-qPCR after repeated MS2-affinity purification, confirmed the RNA sequencing results (24-fold enrichment) and supported the *glpF3* gene as a candidate direct target of sLLM1042+ (Fig. 6F). Notably, no enrichment was detected for the *LLMG_RS04350* transcript (endolysin) mentioned in the previous section (data not shown), suggesting that sLLM1042+ does not form direct contacts with this mRNA under our experimental conditions. To further assess whether the interaction between sLLM1042+ and the candidate *glpF3* mRNA affects translational control, a GFP reporter assay was employed. The fluorescence of the constructed *glpF3-gfp+* translational fusion was measured in a Δ*sLLM1042*+ background. Co-expression of WT sLLM1042+ under the control of a nisin-inducible promoter resulted in an ~1.5-fold reduction in the GFP+ signal, consistent with partial sRNA-dependent translational repression of *glpF3* (Supplementary Fig. S13).

Despite the yet undetermined explicit function of GlpF3, it is annotated as an MIP/aquaporin family protein, most likely participating in the uptake of small uncharged solutes across membranes through the formation of aquaporin-like channels. Bacterial aquaporins can facilitate water, glycerol, urea, glycine, ammonia, and hydrogen peroxide movement, allowing cells to modulate their response to environmental changes and abiotic stresses such as osmotic and salt stress [62]. We hypothesized that the GlpF3 aquaporin may transport ammonium ions due to its gene’s proximity to *LLMG_RS11670*, which encodes the X-prolyl-dipeptidyl-aminopeptidase PepX peptidase. This enzyme enables subsequent amino acid degradation pathways that release ammonium ions, potentially requiring their transport. To investigate a possible role of *glpF3* and its regulation by sLLM1042+, we compared the growth of WT *L. cremoris* MG1363 and the Δ*sLLM1042*+ mutant strain, with or without sRNA complementation, in minimal media supplemented with ammonium sulphate or urea. No growth differences were observed under standard conditions (Supplementary Fig. S14). However, in the presence of 0.001%–0.1% urea and 0.1% ammonium sulphate, the Δ*sLLM1042*+ strain exhibited a slight delay in the onset of logarithmic growth, which was restored upon sLLM1042+ complementation. These findings argue against a major role for *glpF3* in transporting these nitrogen sources under the tested conditions.

The sLLM1042+ region, involved in contacting translation initiation elements on interacting mRNAs, is conserved among *L. cremoris* strains (Supplementary Fig. S9B). We substituted the CCAA sequence with AACC in the sLLM1042+m[30–33] mutant to further explore its role. Mutations in this four-nucleotide stretch, which lies within a longer predicted sRNA–mRNA interaction region (nt 25–60), only slightly altered the regulatory effect compared with the WT sRNA in GFP reporter assay (Supplementary Fig. S13), suggesting that effective modulation of *glpF3* may require a broader interaction region in sLLM1042+. Surprisingly, the resistance to lysozyme conferred by the mutant sRNA was even higher compared to the native sLLM1042+, indicating that the region spanning nucleotides 30–33 appears to be dispensable for regulating this particular bacterial response (Fig. 6G). Given that sLLM1042+ may exert both positive and negative post-transcriptional regulation on different mRNA targets, partial disruption of specific interaction sites could alter the overall regulatory balance. At the same time, the enhanced effect may result from reduced competition with other mRNA targets, indicating that multiple, non-mutually exclusive mechanisms may contribute to the observed phenotype. Conversely, these nucleotide substitutions partially restored sLLM1042+-mediated sensitivity to penicillin G, suggesting that distinct sRNA-interacting regions may be responsible for affecting bacterial responses to different cell wall-targeting antimicrobials, such as lysozyme and penicillin G.

## Discussion

LAB, such as *L. lactis* and *L. cremoris*, have a long history of use for food fermentation and preservation [63]. Their potential in medicine as live drug delivery vehicles, probiotics, and food-grade expression platforms has attracted considerable interest in recent years [64]. However, during fermentation or upon interaction with the human host, bacteria encounter a range of challenging conditions, including antimicrobial stress. In LAB, adaptation to stress is typically a multifactorial process and can involve cross-protection and trade-offs between distinct stress response strategies [65, 66]. In this study, we identified a total of 195 sRNAs in *L. cremoris* MG1363, of which 193 were discovered by sRNA sequencing and two through functional analysis. 36 sRNAs were demonstrated to be responsive to antimicrobial treatment. Further analysis of two sRNAs, sLLM2- and sLLM1042+, suggested that they act as multifaceted regulators, associated with peptidoglycan remodelling, teichoic acid D-alanylation, and effects on known or putative resistance determinants. Although these phenotypes were characterized mainly under conditions of elevated sRNA expression, they nevertheless highlight the complexity of sRNA-mediated regulation and the importance of integrating transcriptomic data with functional validation to harness sRNAs in antimicrobial strategies or strain engineering.

RNA sequencing analysis revealed the expression of 131 previously unannotated sRNAs in *L. cremoris* MG1363. To the best of our knowledge, only one prior study has attempted a genome-wide sRNA identification in this strain [47]. Considering the different experimental approaches and growth conditions employed in the two studies, along with the known environmental sensitivity of sRNA expression, the datasets complement each other, collectively identifying a total of 252 non-redundant sRNAs potentially expressed in *L. cremoris*. Notably, our work expands prior efforts by defining both 5′ and 3′ ends of sRNAs. This provides a more complete resource for future research, improving downstream analysis such as target prediction or reporter design. Interestingly, our functional analysis also uncovered two entirely novel sRNAs that were not initially detected in the global expression dataset, likely reflecting low abundance or condition-specific expression, further underscoring that sRNA discovery highly depends on experimental design, growth phase, and environmental context [67, 68] and that the MG1363 sRNA catalog is likely not yet complete.

We observed that ~10% of sRNAs exhibited differential expression in response to lysozyme or penicillin G exposure. Furthermore, functional assays revealed that elevated expression of selected sRNAs significantly influenced bacterial susceptibility to these antimicrobials. This aligns with previous findings implicating sRNAs in antimicrobial resistance mechanisms. For example, sRNA Rli31, together with RNA-binding protein SpoVG, modulates lysozyme resistance and pathogenesis in the Gram-positive bacterium *Listeria monocytogenes* [69, 70]. Rli31 enhances lysozyme resistance by promoting peptidoglycan modification and remodelling through increased expression of the peptidoglycan deacetylase PgdA and the putative carboxypeptidase PbpX. Our findings reinforce the role of sRNAs as essential elements of bacterial stress response networks, which also extend to antimicrobial resistance mechanisms in both Gram-negative and Gram-positive bacteria [10, 71–73]. Importantly, as the observed sRNA expression changes occurred more than an hour after antimicrobial exposure, this indicates that sRNAs are not only the immediate responders but also a part of a sustained regulatory adaptation. Further insights could be gained by expanding sRNA analyses across multiple time points over extended periods after antibacterial treatment.

Our results support broad effects of both sLLM2- and sLLM1042+, implicating them in bacterial responses to various stresses and suggesting that they act as multifaceted regulators linked to multiple cellular processes. They also point to a connection between sLLM2- sRNA and the susceptibility of *L. cremoris* to lysozyme, linked to changes in teichoic acid D-alanylation. Specifically, we observed that increased levels of sLLM2- are associated with the expression of the *alr* gene, which encodes alanine racemase—an enzyme converting L-alanine to D-alanine. D-alanine is subsequently incorporated into teichoic acids, thus increasing their positive charge and interfering with electrostatic interaction with lysozyme [35]. Beyond resistance to cationic antimicrobials, D-alanylation of teichoic acids in *L. cremoris* has been implicated in protection against autolysis [74] and bacteriophage infection [75]. These traits are beneficial for the probiotic function of bacteria. Moreover, D-alanylation has been shown to be important for gut colonization, as demonstrated in a mouse model using *Lactobacillus reuteri* [76]. Given these findings, it would be valuable to further investigate the role of sLLM2- in modulating D-alanylation and its subsequent effects on other probiotic properties of *L. cremoris*, such as modulation of the host immune response or maintenance of gut barrier integrity. In addition, while some bacteria encode multiple alanine racemase isozymes, *L. cremoris* MG1363 harbours only a single functional *alr* gene, the deletion of which results in D-alanine auxotrophy [77]. As alanine racemase is essential for bacterial viability and absent in humans, it stands out as an attractive target for antibacterial agents. Indeed, a significant number of inhibitors have been developed, many of which show considerable therapeutic potential [78–80]. This suggests that the link between sLLM2- and *alr* may be relevant for antibacterial strategies targeting cell envelope functions.

The results obtained also support the role of sLLM2- in tellurite susceptibility. Tellurite is a highly toxic oxyanion whose antibacterial activity is associated with oxidative stress and broader disruptions of cellular metabolism [81]. In *L. cremoris* multiple loci have previously been implicated in tellurite tolerance, including iron transport (*mntH*), the high-affinity phosphate uptake (*pstA* and *pstD*), putative osmoprotectant transport (*choQ*) genes, and a homolog of the oxidative defence regulator *spx* (*trmA*) [82], as well as thioredoxin TrxD, which plays a role not only in tellurite but also in arsenate detoxification [83]. In this context, our data point to an additional locus—a presumed operon encoding putative tellurium resistance proteins—as increased expression of sLLM2- reduced the abundance of ORF53, TelA, TelB, and TelC proteins, consistent with decreased resistance to tellurite. Although the precise function of these proteins remains uncharacterized, additional roles reported for tellurite resistance genes in broader stress resistance mechanisms [84] suggest that their downregulation can potentially make sLLM2- expressing cells more sensitive to other antimicrobial factors (NaOH, HCl, NaCl, H_2_O_2_). Further direct functional studies will be required to define the contribution of these proteins to tellurite tolerance and broader stress phenotypes. Together, these results support functionally relevant links between sLLM2-, *alr*-associated D-alanine metabolism, and the putative tellurite-resistance operon, although the exact molecular mechanisms remain to be clarified. Similarly, while the MAPS and reporter assays support glpF3 as a candidate target of sLLM1042+, the extent to which this interaction contributes to the antimicrobial phenotypes observed here remains to be resolved.

From an applied perspective, particularly in the context of exploiting endogenous sRNAs to expand the functional capabilities of bacteria [85, 86], our findings, together with previous work, highlight a critical limitation: the identification and selection of candidate sRNAs based solely on sequencing data may be insufficient [87, 88]. While high-throughput transcriptomic analyses provide a valuable foundation for sRNA discovery, functional validation is essential to understand their physiological impact. As demonstrated in this study, overexpression of individual sRNAs can result in divergent phenotypic outcomes ranging from increased or decreased resistance to specific antimicrobial compounds to no measurable effect. Moreover, a single sRNA can modulate susceptibility to multiple stressors, including those with distinct mechanisms of action. For instance, both analysed sRNAs conferred increased resistance to lysozyme while simultaneously heightening sensitivity to penicillin G. This phenotype likely reflects a balance between distinct cell wall defence strategies, such that, in the case of sLLM2-, enhanced teichoic acid D-alanylation, which confers lysozyme resistance, may concomitantly increase sensitivity to β-lactam antibiotics. Notably, a similar inverse relationship between lysozyme and penicillin G susceptibility has already been reported in *L. cremoris* [89, 90], suggesting that such trade-offs may represent a conserved feature of cell wall adaptation relevant to practical sRNA applications.

## Supplementary Material

gkag359_Supplemental_Filess

## Data Availability

The raw proteomics data have been deposited to the Zenodo repository: https://zenodo.org/records/15607078. The sRNA-seq, RNA-seq, and MAPS-seq data are available in the GEO database under the identifier GSE299213. Analysis codes have been deposited at Zenodo: https://doi.org/10.5281/zenodo.15629798.
