# Supplementary material for: Small regulatory RNAs modulate lactococcal susceptibility to cell wall-targeting antimicrobials
Source: Nucleic Acids Res. 2026 Apr 25;54(8):gkag359. doi: 10.1093/nar/gkag359 (PMC13109727; doi:10.1093/nar/gkag359)
Supplement: gkag359_Supplemental_Filess [file gkag359_supplemental_filess.zip › Supplementary Data.pdf]

Supplementary Figures

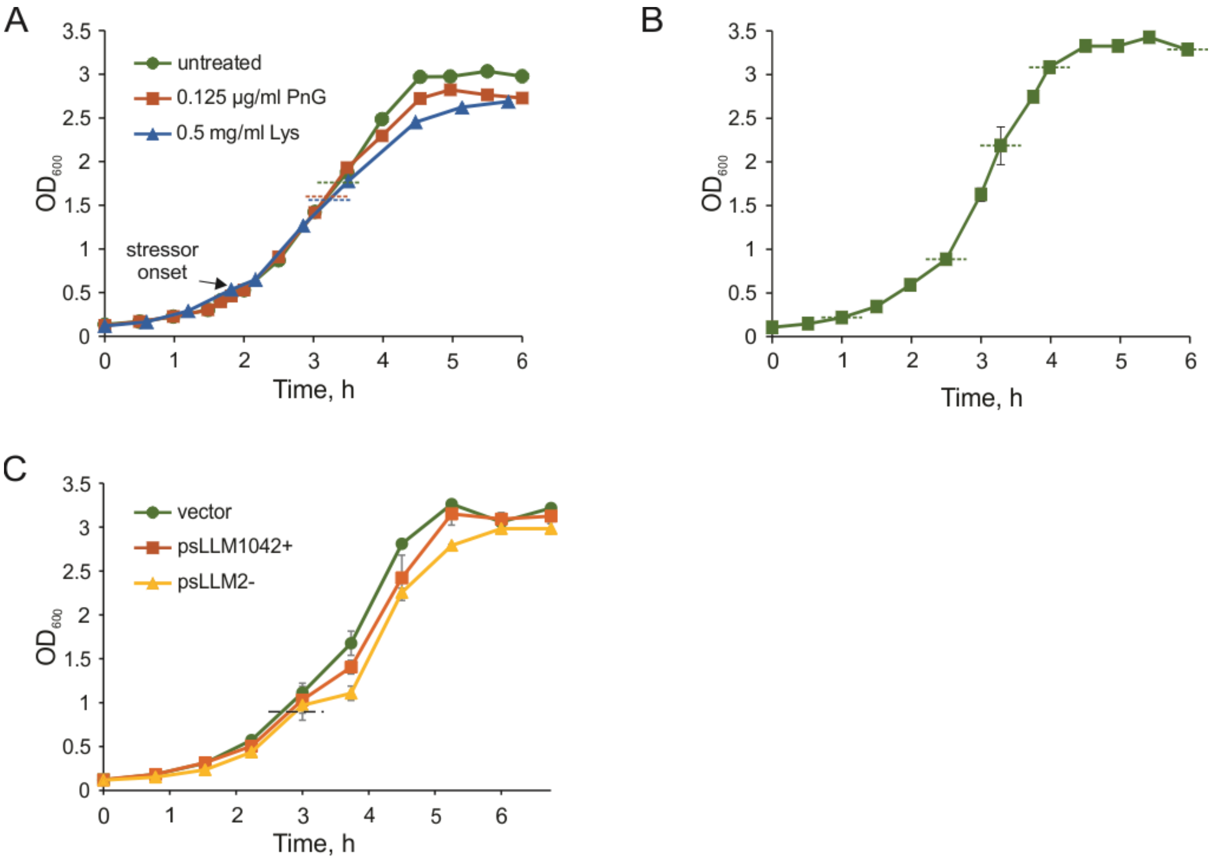

Supplementary Figure S1: *L. cremoris* MG1363 growth curves with growth points at which bacterial biomass was collected for RNA sequencing (marked by dashed lines). (**A**) Bacteria were grown without treatment and with 0.125 µg/ml penicillin G (PnG) or 0.5 mg/ml lysozyme (Lys) added at the indicated time point. (**B**) Bacteria were grown for small RNA analysis at various growth phases. (**C**) Growth curves of bacteria carrying the empty vector or the plasmids containing the sLLM2- or sLLM1042+ genes.

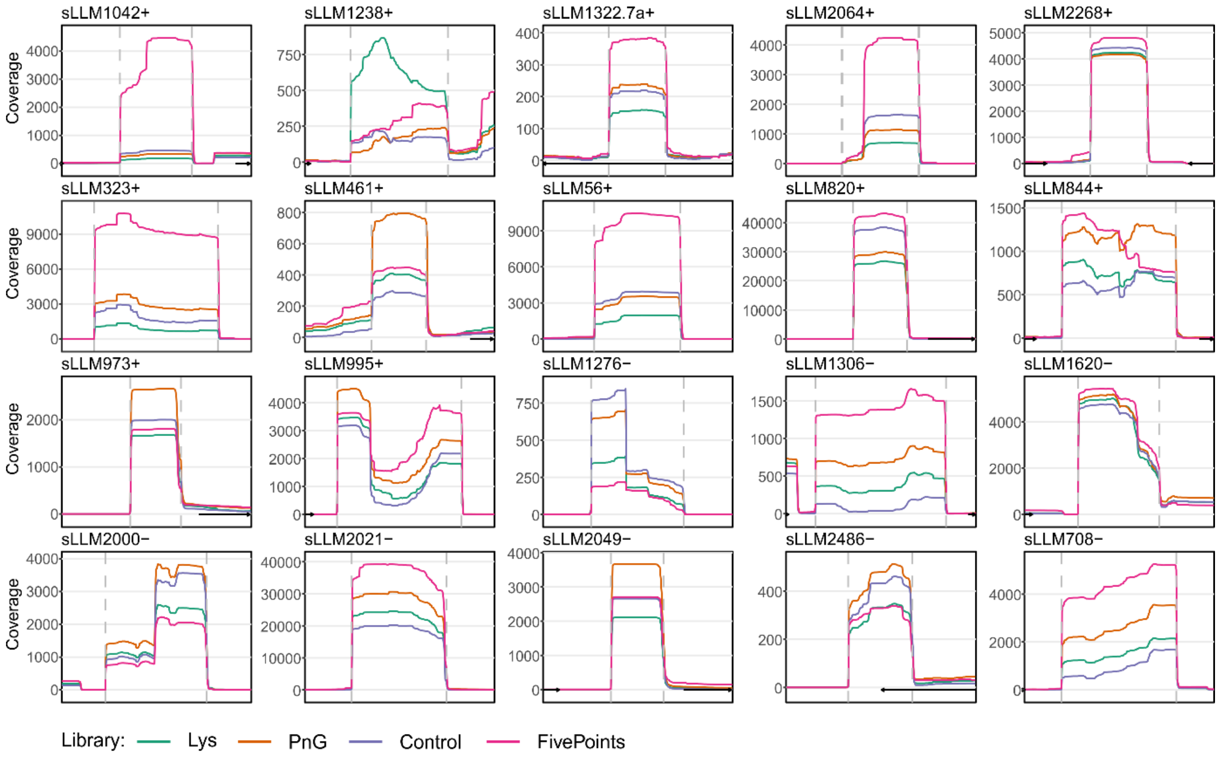

Supplementary Figure S2: Twenty representative plots showing the distribution of the mapped sRNA reads across different RNA-seq libraries. Dashed vertical lines indicate the boundaries of predicted sRNAs; black horizontal arrows represent annotated genes.

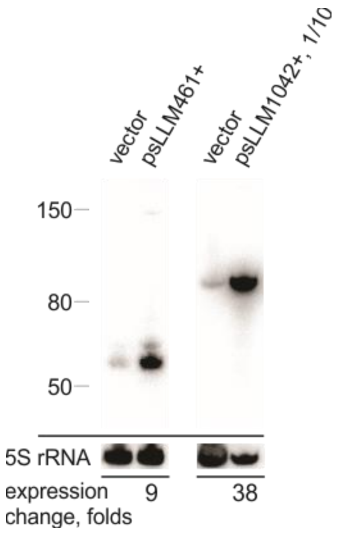

Supplementary Figure S3: Northern blot analysis shows increased levels of sLLM461+ and sLLM1042+ in strains containing the pVE3916 plasmid with cloned sRNA. For psLLM1042+, a tenfold lower amount of total RNA was loaded. 5S rRNA was used as a loading control.

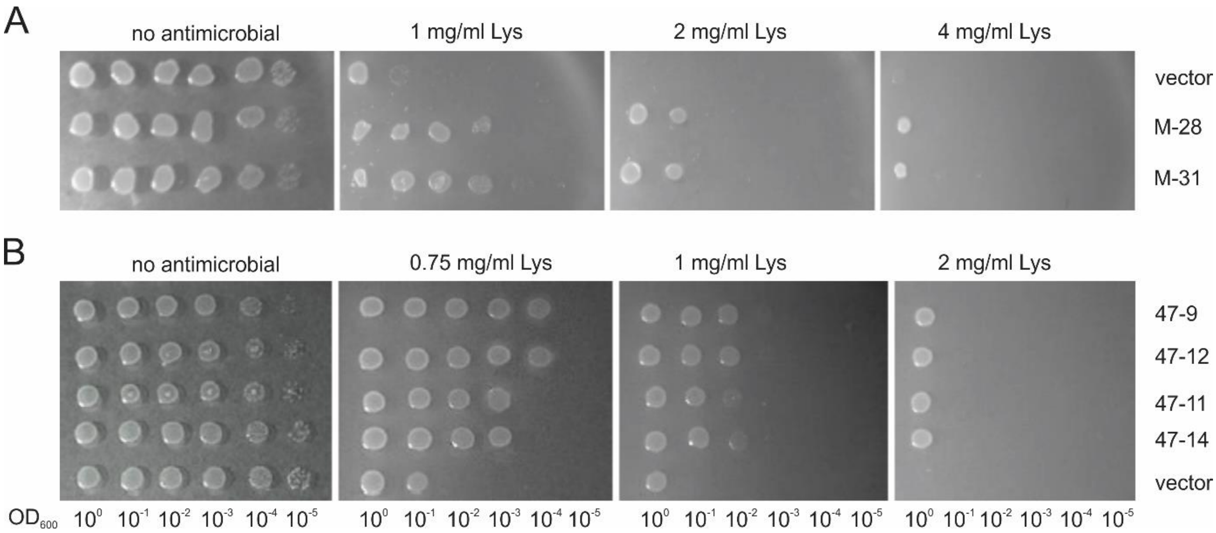

Supplementary Figure S4: Functional analysis of *L. cremoris* genomic fragments selected during the screening on lysozyme plates. (**A**) Plasmids M-28 and M-31 containing an identical genome fragment spanning from 1,075 to 2,075 bp in *L. cremoris* MG1363 *(*NCBI: NC_009004.1) are associated with increased resistance to lysozyme in the wild-type MG1363 strain. (**B**) Plasmids 47-11 and 47-14 (fragment from 1,992,114 to 1,992,972 bp) and 47-09 and 47-12 (fragment from 1,075 to 2,075 bp) enhance lysozyme resistance in the Δ*oatA* strain. Serial dilutions of overnight cultures were spotted onto GM17 agar with the indicated lysozyme concentrations.

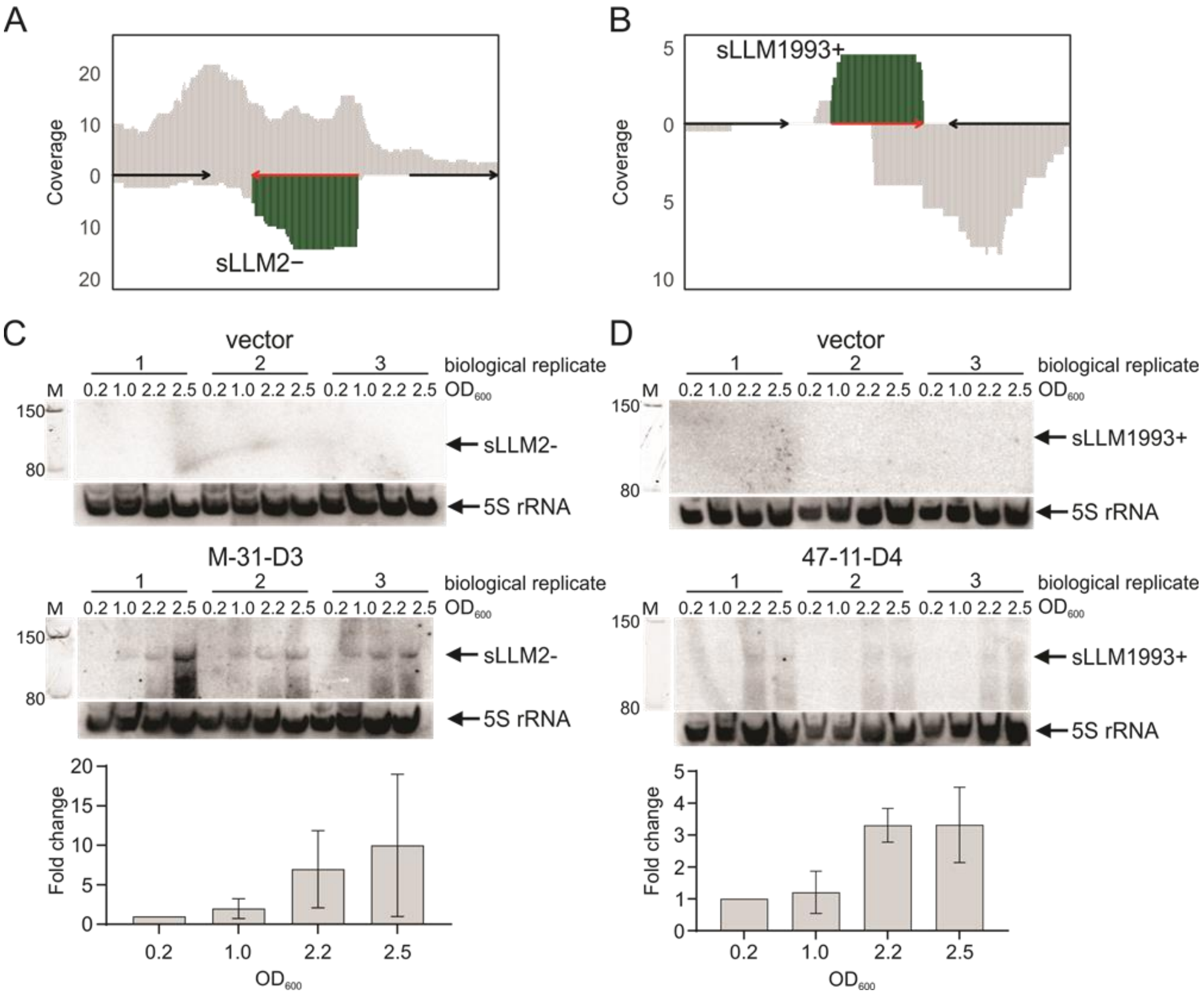

Supplementary Figure S5: The representative plots depicting read coverage in RNA-seq of (**A**) sLLM2-, intergenic sRNA located on the minus strand at positions 1,399-1,480 (BROM and PromoTech predicted promoters at 1,492-1,512 and 1,479-1,518, respectively), and (**B**) sLLM1993+, intergenic sRNA located on the plus strand at positions 1,992,896-1,992,967. (**C**) Northern blot analysis confirms the presence of the sLLM2- sRNA band between the 80 nt and 150 nt RNA marker lines in *L. cremoris* MG1363 carrying M-31-D3 plasmid. (**D**) Northern blot analysis confirms the presence of sLLM1993+ sRNA in cells carrying 47-11-D4 plasmid between 80 nt and 150 nt RNA marker lines. In both cases, due to the low endogenous expression under the tested conditions, the corresponding bands were only detected in strains with exogenous sRNAs. Total RNA was extracted from cells collected at OD_{600} of 0.2, 1.0, 2.2, and 2.5.

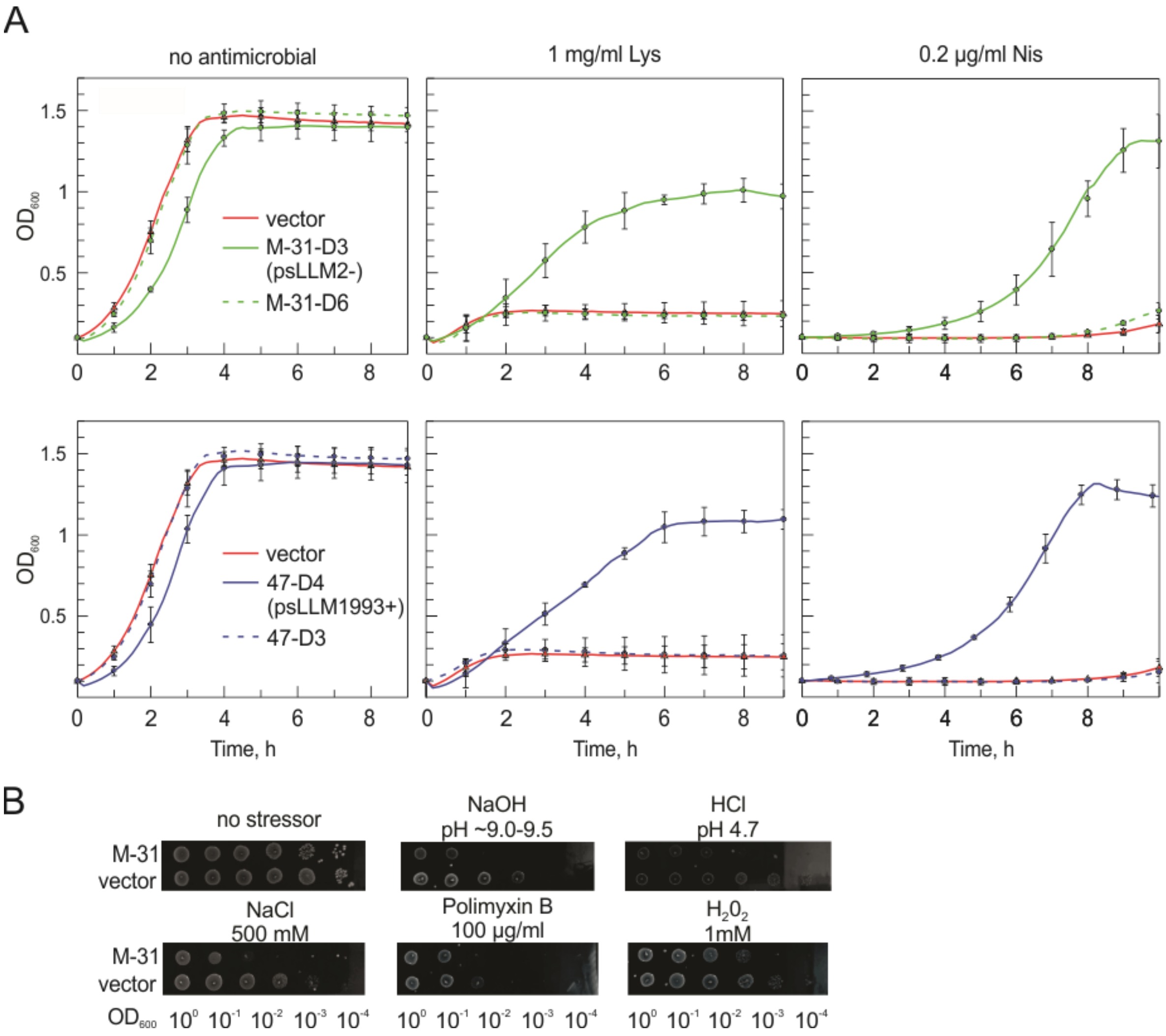

Supplementary Figure S6: Effect of overexpressed intergenic regions from selected fragments M-31 and 47-11 on the growth of *L. cremoris* under different stress conditions. (**A**) Analysis of *L. cremoris* growth in liquid GM17 medium containing specified concentrations of the antimicrobials lysozyme or nisin (Nis) in a 96-well plate. The growth of bacterial cultures, diluted to OD_{600} 0.1, was measured every 10 minutes in triplicate. The green and blue solid lines indicate the growth of antimicrobial-resistant cultures with the M-31-D3 (psLLM2-) or 47-11-D4 (psLLM1993+) plasmids containing intergenic regions from M-31 or 47-11, respectively (see scheme in Figure 2). The green and blue dashed lines represent antimicrobial-sensitive cultures with the 31-D6 and 47-D3 plasmids, respectively, in which the intergenic regions are deleted. The red line represents MG1363 cells carrying the control pVEA1 vector. (**B**) M-31 affects bacterial growth on agar plates with various stressors.

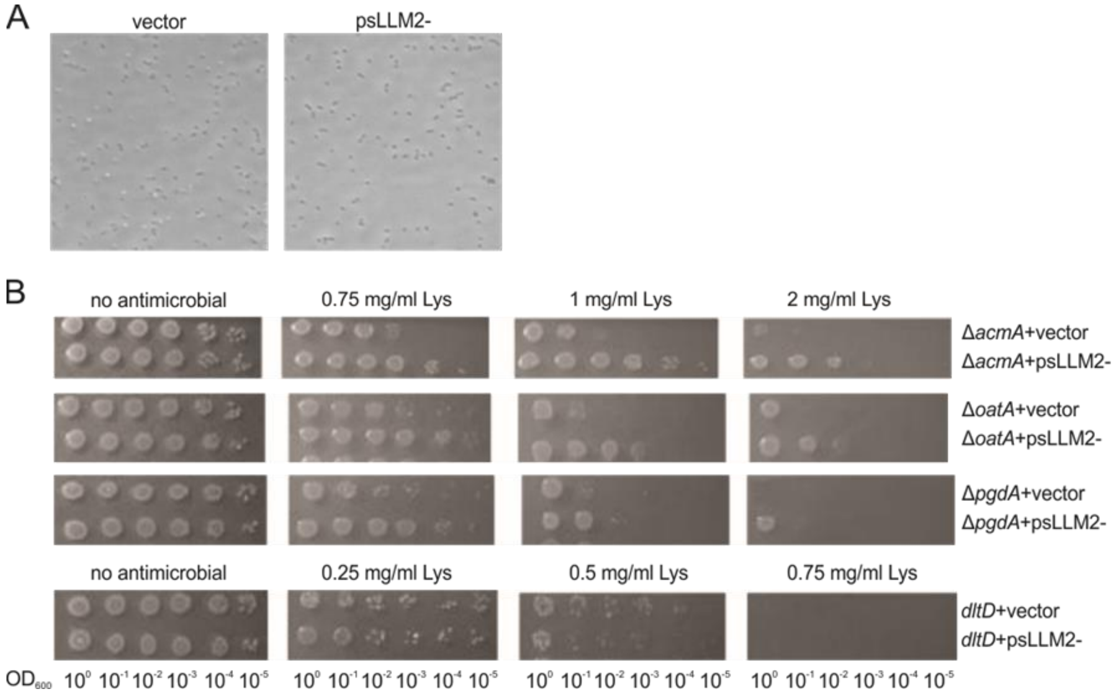

Supplementary Figure S7: Examination of MG1363 and its mutant strains with plasmids carrying sLLM2-. (**A**) Bright-field images of *L. cremoris* cells with pVE3916 vector and psLLM2-. (**B**) Inactivation of *dltD*, but not *acmA*, *oatA,* or *pgdA*, eliminates the increased resistance to lysozyme after the overexpression of sLLM2-.

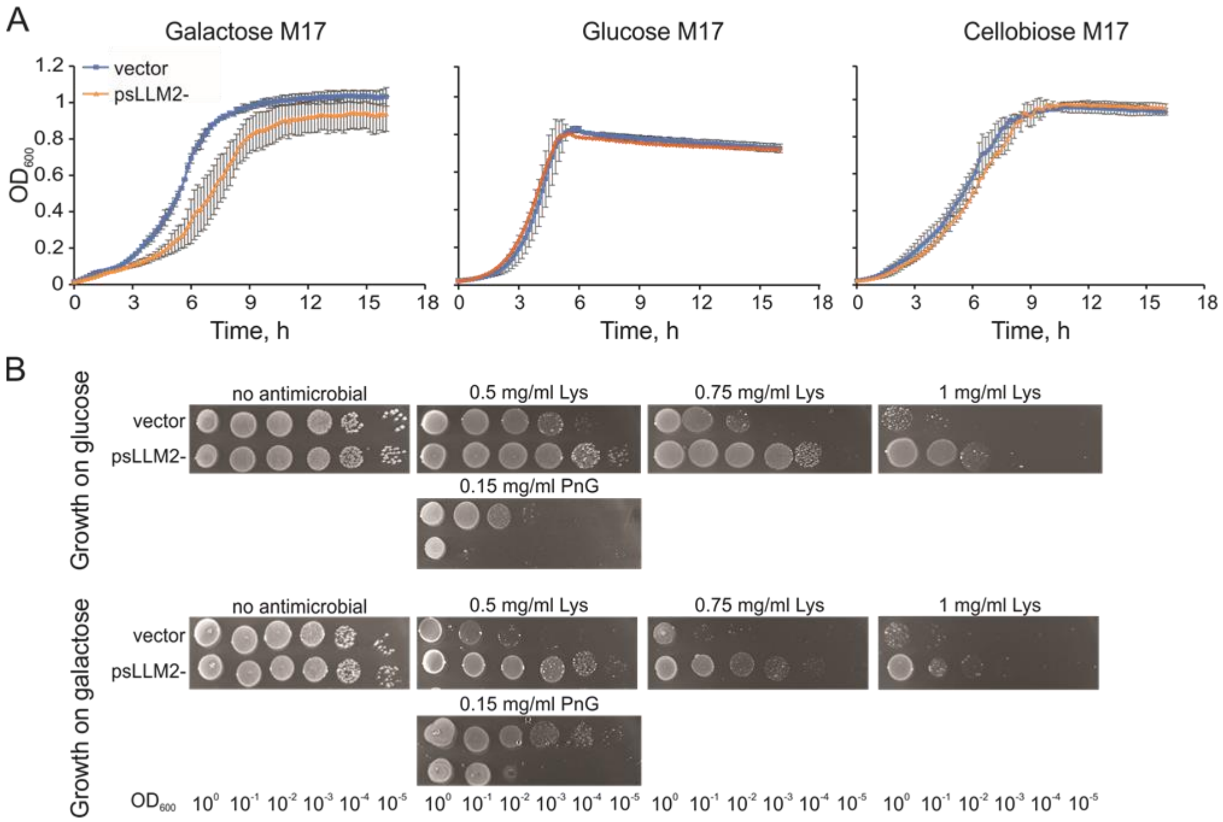

Supplementary Figure S8: Comparison of the effect of sLLM2- on bacterial growth in M17 medium supplemented with different sugars. (**A**) The growth profiles in medium supplemented with galactose, glucose, and cellobiose. (**B**) Gradient dilution drop plate experiment demonstrating the effect of lysozyme and penicillin G treatment.

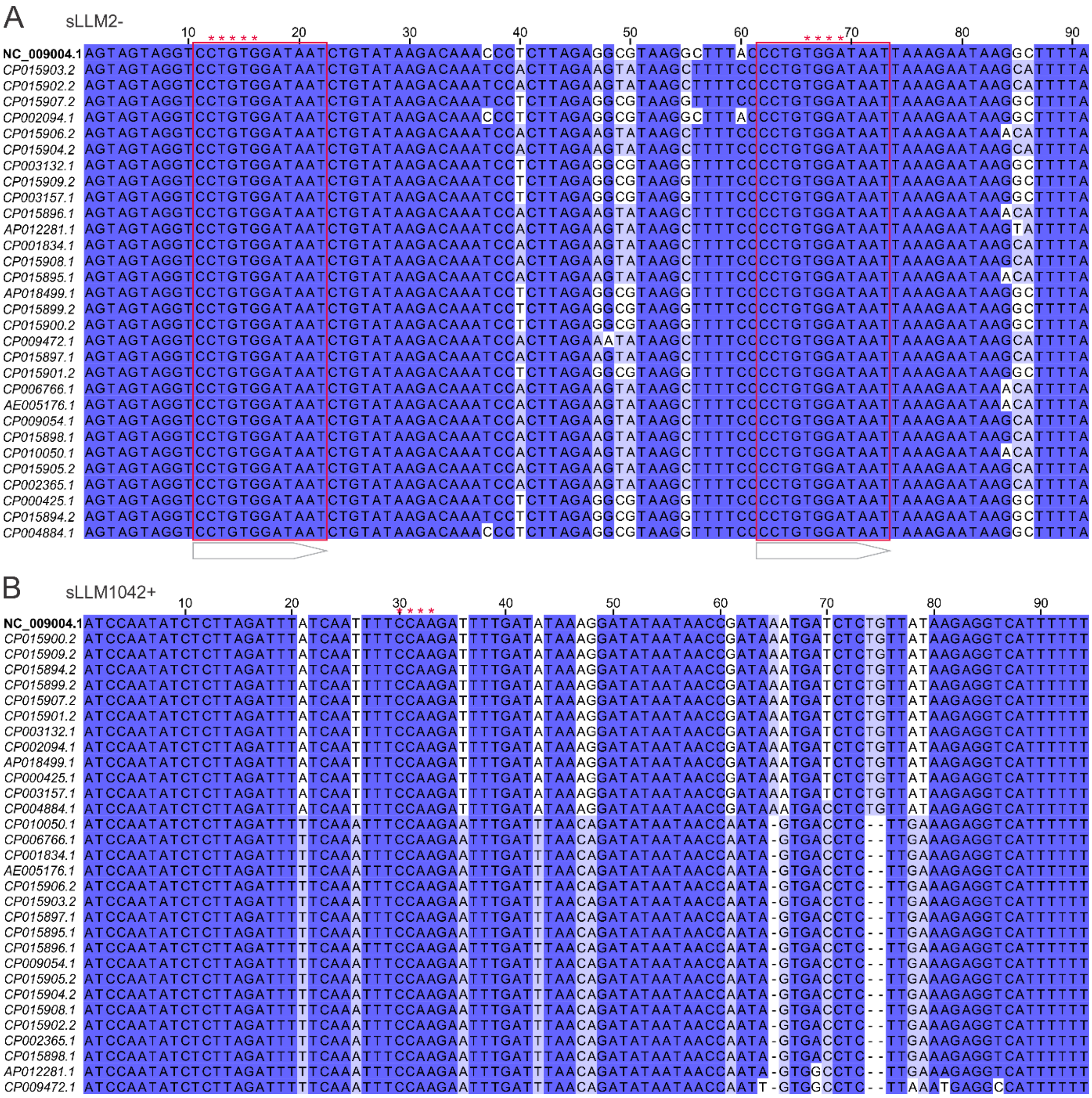

Supplementary Figure S9: Alignment of genomic sequences corresponding to sLLM2- (**A**) and sLLM1042+ (**B**) from MG1363 (top line, NC_009004.1) with the DNA of different *Lactococcus cremoris* and *Lactococcus lactis* strains. Direct 12 nt repeats in sLLM2- are outlined with a red box. Positions of mutated nucleotides are indicated by red stars.

Strains: NC_009004.1 – *L. cremoris* subsp. *cremoris* MG1363, CP015903.2 – *L. lactis* subsp. *lactis* UC08, CP015902.2 – *L. lactis* subsp. *lactis* UC06, CP015907.2 – *L. cremoris* UC109, CP002094.1 – *L. lactis* subsp. *cremoris* NZ9000, CP015906.2 - *L. lactis* subsp. *lactis* UC77, CP015904.2 - *L. lactis* subsp. *lactis* UC11, CP003132.1 - *L. lactis* subsp. *cremoris* A76, CP015909.2 - *L. cremoris* JM4, CP003157.1 - *L. lactis* subsp. *cremoris* UC509.9, CP015896.1 - *L. lactis* subsp. *lactis* 229, AP012281.1 - *L. lactis* subsp. *lactis* IO-1, CP001834.1 - *L. lactis* subsp.

lactis KF147, CP015908.1 - *L. lactis* subsp. *lactis* UL8, CP015895.1 - *L. lactis* subsp. *lactis* 184, AP018499.1 - *L. lactis* subsp. *cremoris* C4, CP015899.2 - *L. cremoris* JM1, CP015900.2 - *L. cremoris* JM2, CP009472.1 - *L. lactis* AI06, CP015897.1 - *L. lactis* subsp. *lactis* 275, CP015901.2 - *L. cremoris* JM3, CP006766.1 - *L. lactis* subsp. *lactis* KLDS 4.0325, AE005176.1 - *L. lactis* subsp. *lactis* Il1403, CP009054.1/71-161 *L. lactis* subsp. *lactis* NCDO 2118, CP015898.1/71-161 *L. lactis* subsp. *lactis* C10, CP010050.1/71-161 *L. lactis* subsp. *lactis* S0, CP015905.2/71-161 *L. lactis* subsp. *lactis* UC063, CP002365.1/71-161 *L. lactis* subsp. *lactis* CV56, CP000425.1/71-161 *L. lactis* subsp. *cremoris* SK11, CP015894.2 - *L. cremoris* strain 158, CP004884.1 - *L. lactis* subsp. *cremoris* KW2.

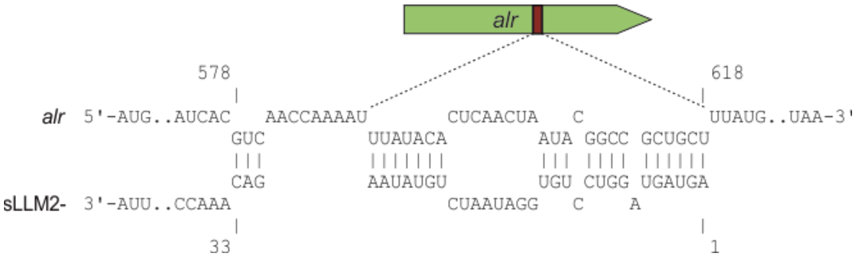

Supplementary Figure S10: The scheme of predicted sLLM2- interaction with *alr* mRNA.

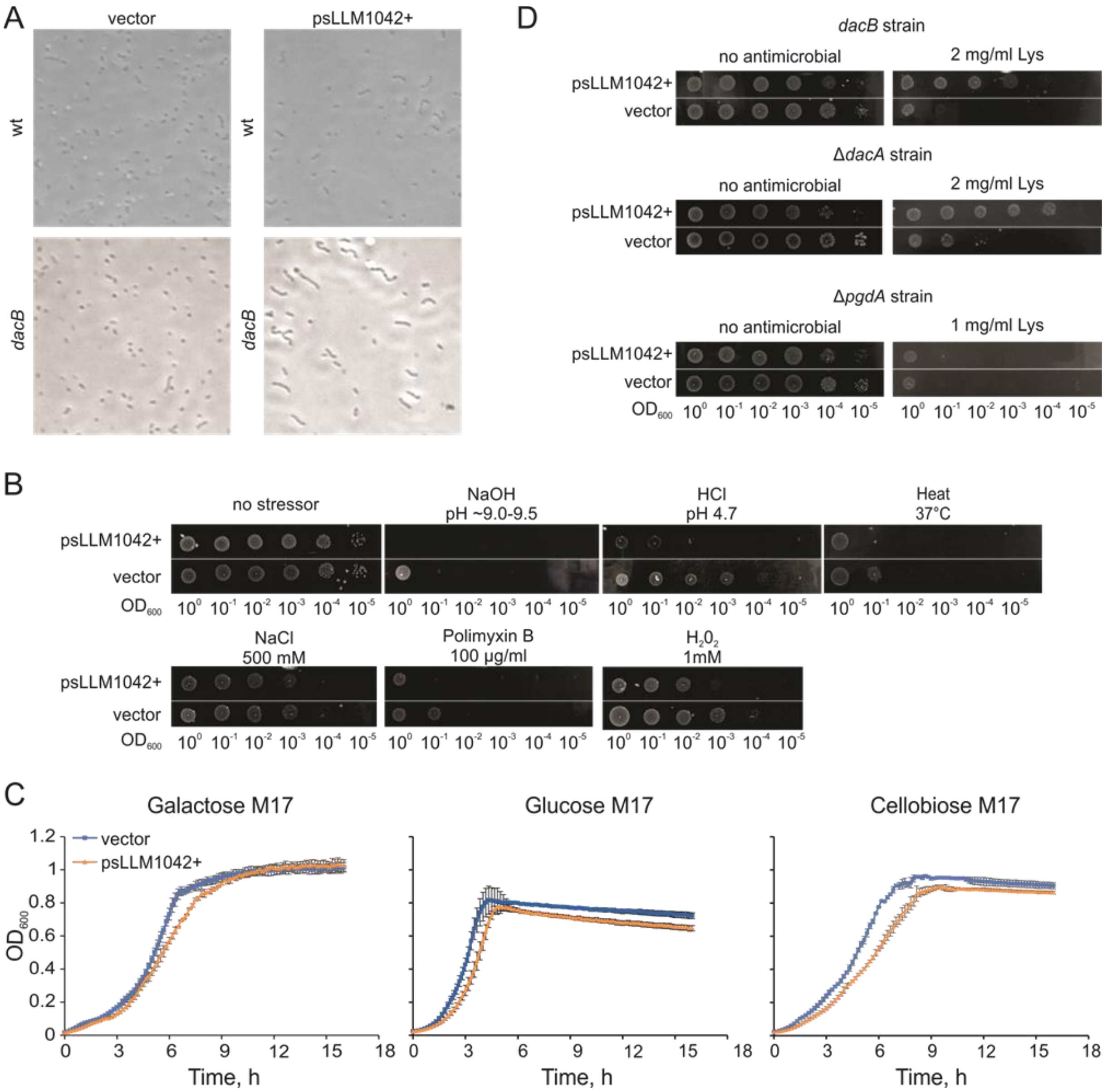

Supplementary Figure S11: Regulatory effects of sLLM1042+ in *L. cremoris*. (**A**) Bright-field images of wild-type and Δ*pgdA* cells with pVE3916 vector and psLLM1042+ plasmid expressing sLLM1042+. (**B**) Influence of sLLM1042+ on bacterial growth on agar plates containing various stressors. (**C**) The growth profiles in medium supplemented with galactose, glucose, and cellobiose. (**D**) Inactivation of the genomic *pgdA*, but not *dacB* or *dacA*, eliminates the increased resistance to lysozyme observed after the sLLM1042+ overexpression.

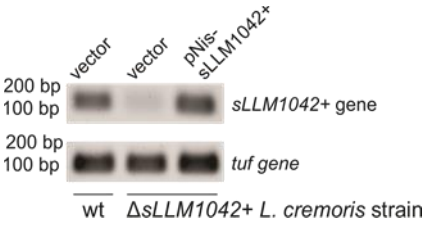

Supplementary Figure S12: Diagnostic PCR confirmed the deletion of the *sLLM1042+* gene in the Δ*sLLM1042+* strain. The *tuf* gene was used as a control.

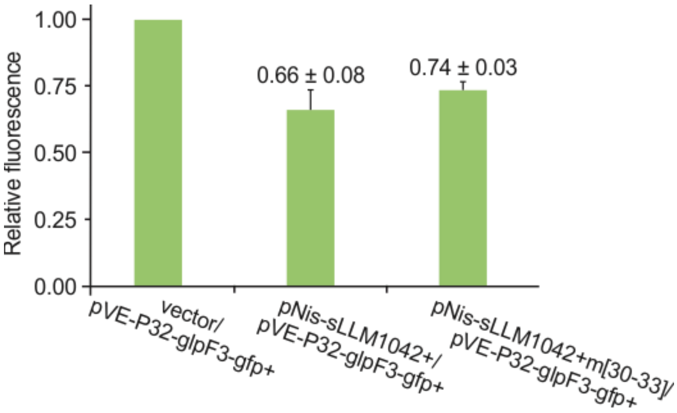

Supplementary Figure S13: GFP reporter assay supports translational modulation of *glpF3* by sLLM1042+, with an approximately 1.5-fold reduction in the GFP+ signal. Translational *glpF3-gfp+* reporter fusion was co-transformed into the *ΔsLLM1042+* strain with either an empty vector or a plasmid expressing sLLM1042+ or sLLM1042+m[30-33] from a nisin-inducible promoter. Cultures were grown overnight with 1 ng/ml nisin and then diluted to OD_{600} 0.1. sRNA expression was further induced with 10 ng/ml nisin, and GFP+ fluorescence was measured in PBS buffer when cultures reached OD_{600} 1.0, using the same cell density for all samples. Fluorescence values were normalized to the empty-vector control set to 1. The data shown represent the mean of two biological replicates.

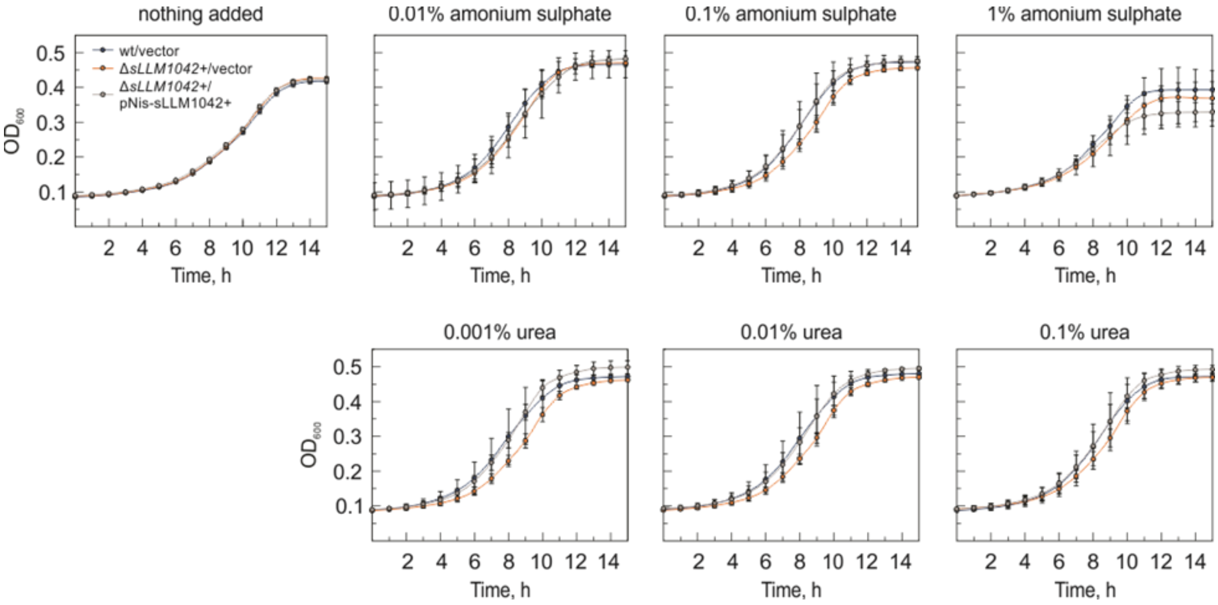

Supplementary Figure S14: Growth analysis of *L. cremoris* in defined minimal liquid medium supplemented with specified concentrations of nitrogen-containing compounds in a 96-well plate format. The blue line indicates growth of the wild-type strain harbouring an empty vector pMSP3545. The orange and grey lines represent the Δ*sLLM1042+* strain carrying either the empty pMSP3545 or pNis-sLLM1042+, respectively. Data represent mean ± SD from three biological replicates.

Supplementary Tables

Table S1: List of bacterial strains used in the current work.

Strain	Genotype	Source
L. cremoris MG1363	Plasmid-free strain	[1]
L. cremoris VES4784 (Δ*oatA*)	MG1363 carrying a deletion of *oatA*	This work
L. cremoris MG1363 Δ*acmA1*	MG1363 carrying a deletion of *acmA*	[2]
L. cremoris VES4534 (Δ*pgdA*)	MG1363 carrying a deletion of *pgdA*	[3]
L. cremoris VES2065 *(dacB*)	MG1363 *dacB:: pRV300*	[4]
L. cremoris VEL1388 (*dltD*)	MG1363 *dltD::*IS*S1*	[5]
L. cremoris NZ9000	MG1363 pepN::nisRK	[6]
L. cremoris NZ9000 (Δ*dacA*)	NZ9000 carrying a deletion of *dacA*	[7]
L. cremoris NZ3900 (Δ*alr*)	NZ3900 carrying a deletion of *alr*	[8]
L. cremoris MG1363 Δ*sLLM1042+*	MG1363 carrying a deletion of *sLLM1042+*	This work
E. coli TG1	F'[*traD36 proAB*$^+$ *lacI*^{q} *lacZ*Δ*M15*] *supE thi-1* Δ(*lac-proAB*) Δ(*mcrB-hsdSM*)5, ($r_K^- m_K^-$)	Lucigen

Table S2: List of plasmids used in the current work.

Name	Source	Purpose
pVE3916	Laboratory collection	Construction of a gene bank of *L. cremoris,* sRNA overexpression
pUC19	Laboratory collection	Construction of pVEA1
pVEA1	This work	Construction of a gene bank of *L. cremoris*
psLLM1042+	This work	sRNA overexpression
psLLM461+	This work	sRNA overexpression
M-31	This work	Selected from the gene bank for conferring lysozyme resistance
M-31-D1	This work	M-31 deletion analysis
M-31-D2	This work	M-31 deletion analysis
M-31-D3 (psLLM2-)	This work	M-31 deletion analysis
M-31-D4	This work	M-31 deletion analysis
M-31-D5	This work	M-31 deletion analysis
M-31-D6	This work	M-31 deletion analysis
47-11	This work	Selected from the gene bank for conferring lysozyme resistance
47-11-D1	This work	47-11 deletion analysis
47-11-D2	This work	47-11 deletion analysis
47-11-D3	This work	47-11 deletion analysis
47-11-D4 (psLLM1993+)	This work	47-11 deletion analysis
psLLM2-m[12-16]	This work	sLLM2- mutant analysis
psLLM2-m[66-69]	This work	sLLM2- mutant analysis
psLLM2-m[12-16][66-69]	This work	sLLM2- mutant analysis
pLCNICK	Gift from Sheng Yang	Construction of Δ*sLLM1042*+ strain
pVE-P32	This work	Construction of Δ*sLLM1042*+ strain
pLCNICK_P32	This work	Construction of Δ*sLLM1042*+ strain
pMSP3545	[9]	MAPS experiment, GFP reporter assay
pNis-sLLM1042+	This work	MAPS experiment, GFP reporter assay
pNis-2xMS2-sLLM1042+	This work	MAPS experiment
phage – ubc – nls – 2xmcp – egfp – BirA*	Addgene	MAPS experiment
pGEX-5X-1	Laboratory collection	MAPS experiment
pGEX-5X-1-2xMCP	This work	MAPS experiment
psLLM1042+m[30-33]	This work	sLLM1042+ mutant analysis
pZEP16	[10]	GFP reporter assay
pVE-P32-glpF3-GFP+	This work	GFP reporter assay
pNis-sLLM1042+m[30-33]	This work	GFP reporter assay

Table S3: List of *L. cremoris* 5S rRNA-specific probes used for rRNA depletion. +T – LNA nucleotide, R – G or A nucleotide at a particular position, Biotin-TEG – biotin attached to the 3' end of a DNA oligonucleotide through the TEG spacer. LNA containing oligonucleotide was synthesized at Exiqon, and the rest were purchased from Metabion.

Probe	Sequence 5'-3'
5S comp. 1	T+TCTGTGTTCGRCA+TGGGAACAGG+TGTATC-Biotin-TEG
5S *L. cremoris* comp 2	TACTTCCGCCGTAGATGGACTTAAC-Biotin-TEG
5S *L. cremoris* comp 3	GGCCACTCGCATATCTCCCAGG-Biotin-TEG

Table S4: List of DNA oligonucleotides used for Northern blot analysis.

Probe	Sequence 5'-3'
sLLM56+	GTTAGACTGACCTTTTATTGTGGTTGTC
sLLM323+	GCTCTCTGTACTGACTTTCACAATTACT
sLLM461+	CAGAGGGGAGCCTACCGTTACTGATTT
sLLM708-	GCTTTCACCAAACGCAAGCTCTCTAAA
sLLM820+	TGTCTGTCCTTAATGGATACGAGGATGT
sLLM844+	AAGACAGTCTGTAGGATTTAACCATACA
sLLM973+	GGTGCAAAAGTCCGAATTCCTATTGGAAAG
sLLM995+	CTCTGATGAATTTATCTGCTCGCACAACCGCAGACT
sLLM1042+	ACAGAGATCATTTATCGGTTATTATATCCT
sLLM1238+	ACGTATGGAGTTCTTAATCGGCAGGTAC
sLLM1276-	CAACTTAATGTTTAACTATAAAGACTTATTGGGC
sLLM1306-	TAAGTCTTCTGATTCCAAAAGGACAAACAG
sLLM1322.7a	ACACAAAAGGAACTTGCAGAGCTTGCC
sLLM1620-	GATTTTCAATAGACCTTACCTCATTCATT
sLLM2000-	ACTGTTGATTGTTGTGTTTTCATGCGGAC
sLLM2021-	TCCCGAGCTTTGACCATCGAGTTCTTATCT
sLLM2049-	CAAGACGTATTAAGCTTGATTTTTATTTTCTTTTAAATG
sLLM2064+	CAACTAGAGGATGATGAGTGTTAGTACA
sLLM2268+	GAGTGTGACTTGAATCAAAGGGGTTTATG
sLLM2486-	CCAACCGCTACCAACGAAAAAGGC
sLLM2-	TCTTATACAGATTATCCACAGGACCTACTAC
sLLM1993+	TTTATATGACTGCCTCTTTAATCCGTG
5S rRNA	TACTTCCGCCGTAGATGGACTTAACTTCTG

Table S5: List of DNA oligonucleotides used for cloning.

Oligonucleotide	Sequence 5'-3'
sLLM1042+_Fw	CTTTAGCCAGAAGAAGACAAAATAGTTC
sLLM1042+_Rv	TTCCATCATTATTATCCTTACATCTCTC
sLLM461+_Fw	TCTGGCAATTTCGTCATTAAAATCTACTG
sLLM461+_Rv	CTTAGTTGACATCTAAATCTCCTCTTATC
M-31-tg-dir	CAGTTTAAGCTTTTGAAAATTTAATCATGATA
M-31-tg-rev	ATATTGCTCGAGTTAAGCGTAAATTTTAGAAC
M-31–Δtg–dir	CACAGTTCTAAAATTTACGCTTAATGC
M-31–Δtg–rev	GGACCTACTACTATTACTATTATATTT
47-11-tg-dir	AGGTTAAGCTTAATATTCAATCATAAATTCTAACC
47-11-tg-rev	TAATGCTCGAGATTATAAATATATAAACAGTAATA
sLLM2-mut_Fw1	AAGCAGACAAGTAAGCCTC
sLLM2-mut_Rv1	GACCTACTACTATTACTATTATATTTATA
sLLM2-mut_Fw2	GACACGATAATCTGTATAAGACAAACCC
sLLM2-mut_Rv2	AAGCATCTTACGGATGGC
sLLM2-mut_Fw3	TAATTAAAGAATAAGGCTTTTATAAGTCC
sLLM2-mut_Rv3	AGGTCAGGGTAAAGCCTTACG
sLLM2-mut_Fw4	GATAATCTGTATAAGACAAACCC
sLLM2-mut_Rv4	GTGTCGACCTACTACTATTAC
F_invert_plasmid	TTATAAGGAGGCACTCACC
R_invert_plasmid	AAATCATTGTATCTAACAAACTTC
sLLM1042+_Fw2	CATCCAATATCTCTTAGATTTATCAAT
R_LLM_1042_PaeI	GTTGGCATGCCCTTACATCTCTCTATTTCTGAC
R_invert_MS2_plasmid	CAGACCCTGATGGTGTCTGAAAAACGTACCCTGATGGTGTACGAAATCATTGT ATCTAACAAACTTC
sLLM1042+mut_Fw1	ATTCTCTGGTATTTGGACTCC
sLLM1042+mut_Rv1	GGTTAAAATTGATAAATCTAAGAGATATTG
sLLM1042+mut_Fw2	GATTTTGATATAAAGGATATAATAACCG
sLLM1042+mut_Rv2	AAACAAGTAGCGAAAACTCG
Glp3 dir	GGTTATTATATTAAAATCAAATTTTGGAGGGTTCC
glp-GFP+ rev	CTTCTCCTTTACGTCCAACAAATTCAGTGA
glp-GFP+ dir	TCACTGAATTTGTTGGACGTAAAGGAGAAG
GFP+ rev	CAATCGATTTATTTGTAGAGCTCATCCATGCC
sLLM1042+ Dir	CATCCAATATCTCTTAGATTTATCAATTTTCC
sLLM1042+ Pae Rev	TGGCATGCCCTTACATCTCTCTATTTCTGACCAT

Table S6: List of DNA oligonucleotides used for RT-qPCR analysis.

Probe	Sequence 5'-3'
arcA_Fw	ATTGCGTGACAATGGTGCTG
arcA_Rv	CTGCTTCATGCAACAAATGGC
arcC1_Fw	GCCGTGGTTATCGTCGAGT
arcC1_Rv	CTCCGCCAGAAGCAATCAG
arcC2_Fw	ATCGGTCCATTCTACGACGA
arcC2_Rv	GACGATAGCCACGACCTGAG
arcD1_Fw	ACAGGTCAACCTGGTTTGGT
arcD1_Rv	TCCATGACAACCAAGCTCCA
argF_Fw	GGCATCCAACACAAATGATTGC
argF_Rv	TGTTACGACCATCACCAACG
cluA_Fw	TTCCGAGCAGGTCAGAATCC
cluA_Rv	AAGTCCTGTCATTCCGTCGC
cpdC_Fw	TCACAGGACATCAGCATCGG
cpdC_Rv	TCCCCAACAAAATCCCCTCG
guaC_Fw	TTGCTCATGGTCACGCAGAC
guaC_Rv	AGCTCACGAACAGCTTCTGG
llmg_0439_Fw	ACTGACGAGTCTTCGGTCAA
llmg_0439_Rv	AGGTCAGGACAAAATCGCTCA
llmg_0760_Fw	TCATGTTTATTGGGGGCGGA
llmg_0760_Rv	CACGAGCGTCTCCATTACCG
llmg_2513_Fw	GCTTCTTGTTGGACTTGCCG
llmg_2513_Rv	GCTTGACCAAATGGTGCGAC
noxE_Fw	CAAATCGTAACCAACAAGGCGA
noxE_Rv	CACTTGCCAAGGCACTGTTG
pbuO_Fw	CTTGGCTCTTTTGACTGGCG
pbuO_Rv	GCTGCACCAAAGGTTGTTCC
ps350_Fw	GGAAGATGATGGACGTGGCA
ps350_Rv	AGCATCACGAAAAGCACCTG
ps356_Fw	TCGGAGGAACTTACGGTGGA
ps356_Rv	GGGCTCGTTTACCTTGAGCA
purC_Fw	GCTGCTTTATGAAGGAAAAGCC
purC_Rv	AAGCTGTCGCTTGATCACAA
rcfB_Fw	TTATGTTGCGGAGCCAGACT
rcfB_Rv	GCTGTTGGAGACTCAATTCGT
ribA_Fw	TGCATTTGTCAGCAATGACGG
ribA_Rv	ACGGTCAAAGGCTGATACCC
ribD_Fw	TGACCTTAGAACCTTGTGCCC
ribD_Rv	AGACTCCCAATGACTACACGC
ribU_Fw	TGGGATTGTCTTGGCGACTA
ribU_Rv	GCAGCCAGACGATACAATGG
tuf-Fw	CACTCCATTCTTCGACAACTACC
tuf-Rv	AGGCATTACCATTTCAGTTCCTT

xpt_Fw	CGGTAAGCGTTTTGCACAAGT
xpt_Rv	GCAGCATATAAAGCAGGGGC
sLLM2-_Fw	GATAATCTGTATAAGACAAACCC
sLLM2-_Rv	CAGGGTAAAGCCTTACGCCTCTAAG
alr_Fw	TGGGTCGGATTGGTATTCGTG
alr_Rv	CCGCAGTCGCAAAATGAGTG
sLLM1042+_Fw1	CATCCAATATCTCTTAGATTTATCAAT
sLLM1042+_Rv1	ACAGAGATCATTTATCGGTTATTATATCCT
glpF3_Fw	GGTACAAAAGCTCACGCACA
glpF3_Rv	AAAAAGTCCTGAGGCTGCAA

Table S7: List of DNA oligonucleotides used for the construction of the Δ*sLLM1042+* strain.

Oligonucleotide	Sequence 5'-3'
pVE_seq_primerF	CCCGTTAGTTGAAGAAGGTT
pVE_seq_primerR	GCTACGATAACGCCTGTTTT
1042Cs9upF	AAGTGGCACCGAGTCGGTGCTTTTTTTGAGGCCAAAGCGACAAAAGTAG
1042Cs9upR	TATCATATTTTCAATAAGATAATTCTATCTATTTTCAAGTG
1042Cs9downF	AGATAGAATTATCTTATTGAAAATATGATATAATAATGGTCAG
1042Cs9downR	GCTACGGATCGCATCTTTTTCTAAACTAGGGCCATGTCAACAAAGCCGGTG
1042sgRNA	AGGATGATATCACCTCTAGTATCCTTTATATCAAAATCTGTTTTAGAGCTAGAAATA
gTEMdnMOD	CTCAAAAAAAGCACCGACTC
R_LLM_1042_PaeI	GTTGGCATGCCCTTACATCTCTCTATTTCTGAC
1042checkF	GCGATGCTTGCGGTTAAAGA
F_invert_pVE_P32	AGTATAGCATTTTGTGAAGTTTTTTTCTAGTCCACAGCATGTTCAATGATGTCG
R_invert_pVE_P32	AGGTAGGTAAAAAAATATTCGGAGGAATTTTGCATGCATTGAGAAGATTGCCGAAA

Table S8: Expression changes of selected RNAs in cells with elevated sLLM2- levels, identified by RNA-seq and verified by RT-qPCR analysis. *p < 0.05, **p < 0.01, ***p < 0.0001.

NCBI locus tag	Gene	Fold change, RNA-seq	Fold change (range of dispersion), RT-qPCR
		Genes associated with riboflavin synthesis	
LLMG_RS07710	*ribD*	28.8	32.8 (26.0 – 40.0)***
LLMG_RS07700	*ribA*	37.2	46.6 (36.8 – 57.3)***
LLMG_RS06050	*ribU*	5.5	7.9 (5.4 – 10.7)***
		Genes associated with purine metabolism	
LLMG_RS09575	*pbuO*	9.7	9.2 (6.8 – 11.7)***
LLMG_RS07130	*guaC*	8.4	8.1 (5.9 – 10.5)***
LLMG_RS04950	*purC*	21.4	38.7 (32.0 - 45.9)***
LLMG_RS06820	*xpt*	3.4	3.4 (3.2 – 3.5)***
LLMG_RS01640	*cpdC*	3.6	4.7 (2.8 – 6.9)**
		Putative transglycosylase	
LLMG_RS03925	*llmg_0760*	2.4	2.1 (1.6 – 2.6)**
		NADH oxidase	
LLMG_RS02085	*noxE*	-4.4	-6.9 (5.9 – 8.3)***
		SspB-related isopeptide-forming adhesin	
LLMG_RS07065	*cluA*	-2.8	-2.4 (2.0-3.1)*

Table S9: Expression changes of selected RNAs in cells with elevated sLLM1042+ levels, identified by RNA-seq and verified by RT-qPCR analysis. *p < 0.05, **p < 0.01, ***p < 0.0001.

NCBI locus tag	Gene	Fold change, RNA-seq	Fold change (range of dispersion), RT-qPCR
		MFS transporter	
LLMG_RS12625	*llmg_2513*	391	241 (199 – 286)***
		Crp/Fnr family transcriptional regulator	
LLMG_RS12620	*rcfB*	4.1	3.4 (2.6 – 4.4)***
		Genes associated with arginine/ornithine metabolism	
LLMG_RS11595	*arcA*	3.2	2.7 (2.7 – 3.1)***
LLMG_RS11590	*argF*	3.4	2.3 (2.1 – 2.6)***
LLMG_RS11580	*arcC1*	3.5	2.6 (2.0 – 3.2)*
LLMG_RS11575	*arcC2*	3.5	3.2 (2.55 – 3.81)**
LLMG_RS11585	*arcD1*	3.3	2.6 (2.07 – 3.18)*
		Phage tail protein	
LLMG_RS04320	*ps350*	-2.8	-2.1 (1.4 – 4.2)*
		Putative endolysin	
LLMG_RS04350	*ps356*	-3.2	-2.70 (2.0 – 3.8)***
		MurR/RpiR family transcriptional regulator	
LLMG_RS02235	*llmg_0439*	-2.2	-6.9 (5.9 – 8.3)***
		SspB-related isopeptide-forming adhesin	
LLMG_RS07065	*cluA*	2.0	2.1 (1.4 – 3.2)*

Supplementary Notes

sRNA nomenclature

Names for *L. cremoris* sRNAs were assigned using a modified BSRD-style nomenclature. Each entry begins with "**s**" (**s**RNA), and is followed by the three-letter genome identifier **LLM** for *Lactococcus cremoris* subsp. *cremoris* MG1363 (formerly classified as ***L. l****actis* subsp. *cremoris* **M**G1363) as abbreviated in KEGG. The next numeric block encodes genomic position – **start coordinate/1000 + 1 (kilobases)**, and a trailing "**+**"/"**–**" denotes the coding strand. When multiple sRNAs occur within the same kilobase on the same strand, we append "**.x**" to indicate the hundred-base sub-interval (x = 1–10). For example, a plus-strand sRNA starting at 460,910 bp is named sLLM461+, and plus-strand sRNAs starting at 1,208,748 bp and 1,208,841 bp are named sLLM1209.8+ and sLLM1209.9+, respectively. When several genomic loci encode sRNAs with identical sequences, we distinguish them by adding a lowercase letter immediately before the strand symbol, assigning "**a**" to the first copy and subsequent letters in alphabetical order (e.g., sLLM546a+, sLLM546b+).

REFERENCES

1. Gasson MJ. Plasmid complements of Streptococcus lactis NCDO 712 and other lactic streptococci after protoplast-induced curing. *J Bacteriol* 1983;**154**:1–9.

2. Buist G, Kok J, Leenhouts KJ *et al.* Molecular cloning and nucleotide sequence of the gene encoding the major peptidoglycan hydrolase of Lactococcus lactis, a muramidase needed for cell separation. *Journal of Bacteriology* 1995;**177**:1554–63.

3. Veiga P, Bulbarela-Sampieri C, Furlan S *et al.* SpxB Regulates O-Acetylation-dependent Resistance of Lactococcus lactis Peptidoglycan to Hydrolysis. *Journal of Biological Chemistry* 2007;**282**:19342–54.

4. Courtin P, Miranda G, Guillot A *et al.* Peptidoglycan Structure Analysis of Lactococcus lactis Reveals the Presence of an l,d-Carboxypeptidase Involved in Peptidoglycan Maturation. *Journal of Bacteriology* 2006;**188**:5293–8.

5. Duwat P, Cochu A, Ehrlich SD *et al.* Characterization of Lactococcus lactis UV-sensitive mutants obtained by ISS1 transposition. *J Bacteriol* 1997;**179**:4473–9.

6. Kuipers OP, de Ruyter PGGA, Kleerebezem M *et al.* Quorum sensing-controlled gene expression in lactic acid bacteria. *Journal of Biotechnology* 1998;**64**:15–21.

7. Roces C, Courtin P, Kulakauskas S *et al.* Isolation of Lactococcus lactis mutants simultaneously resistant to the cell wall-active bacteriocin Lcn972, lysozyme, nisin, and bacteriophage c2. *Appl Environ Microbiol* 2012;**78**:4157–63.

8. Steen A, Palumbo E, Deghorain M *et al.* Autolysis of Lactococcus lactis is increased upon D-alanine depletion of peptidoglycan and lipoteichoic acids. *J Bacteriol* 2005;**187**:114–24.

9. Bryan EM, Bae T, Kleerebezem M *et al.* Improved vectors for nisin-controlled expression in gram-positive bacteria. *Plasmid* 2000;**44**:183–90.

10. Hautefort I, Proença MJ, Hinton JCD. Single-Copy Green Fluorescent Protein Gene Fusions Allow Accurate Measurement of Salmonella Gene Expression In Vitro and during Infection of Mammalian Cells. *Applied and Environmental Microbiology* 2003;**69**:7480–91.
